# Multi-Disciplinary Monitoring Networks for Mesoscale Underground Experiments: Advances in the Bedretto Reservoir Project

**DOI:** 10.3390/s23063315

**Published:** 2023-03-21

**Authors:** Katrin Plenkers, Andreas Reinicke, Anne Obermann, Nima Gholizadeh Doonechaly, Hannes Krietsch, Thomas Fechner, Marian Hertrich, Karam Kontar, Hansruedi Maurer, Joachim Philipp, Beat Rinderknecht, Manuel Volksdorf, Domenico Giardini, Stefan Wiemer

**Affiliations:** 1 Department of Earth Sciences, ETH Zurich, 8092 Zurich, Switzerland; 2 Nagra, 5430 Wettingen, Switzerland; 3 Swiss Seismological Service, ETH Zurich, 8092 Zurich, Switzerland; 4 Geotomographie, 56567 Neuwied, Germany; 5 Solexperts, 8617 Moenchaltorf, Switzerland; 6 Gesellschaft für Materialprüfung und Geophysik mbH (GMuG), 61231 Bad Nauheim, Germany; 7 BRtechnik, 8713 Zurich, Switzerland; 8 Drillwerk, 30457 Hanover, Germany

**Keywords:** in situ rock experiments, geothermal exploration research, Bedretto Underground Laboratory for Geosciences and Geoenergies, multi-discipline monitoring, fiber-optic monitoring, microseismic monitoring, ultrasonic monitoring

## Abstract

The Bedretto Underground Laboratory for Geosciences and Geoenergies (BULGG) allows the implementation of hectometer (>100 m) scale in situ experiments to study ambitious research questions. The first experiment on hectometer scale is the Bedretto Reservoir Project (BRP), which studies geothermal exploration. Compared with decameter scale experiments, the financial and organizational costs are significantly increased in hectometer scale experiments and the implementation of high-resolution monitoring comes with considerable risks. We discuss in detail risks for monitoring equipment in hectometer scale experiments and introduce the BRP monitoring network, a multi-component monitoring system combining sensors from seismology, applied geophysics, hydrology, and geomechanics. The multi-sensor network is installed inside long boreholes (up to 300 m length), drilled from the Bedretto tunnel. Boreholes are sealed with a purpose-made cementing system to reach (as far as possible) rock integrity within the experiment volume. The approach incorporates different sensor types, namely, piezoelectric accelerometers, in situ acoustic emission (AE) sensors, fiber-optic cables for distributed acoustic sensing (DAS), distributed strain sensing (DSS) and distributed temperature sensing (DTS), fiber Bragg grating (FBG) sensors, geophones, ultrasonic transmitters, and pore pressure sensors. The network was realized after intense technical development, including the development of the following key elements: rotatable centralizer with integrated cable clamp, multi-sensor in situ AE sensor chain, and cementable tube pore pressure sensor.

## 1. Introduction

In situ experiments have become a backbone in geoscientific research as they close the gap between laboratory experiments and natural, full scale observations. Especially the fields of earthquake nucleation [1,2,3,4,5], nuclear waste deposition [6,7,8], and various aspects of the engineering or structural health monitoring of underground structures such as tunnels, mines, or caverns [9,10] are investigated using in situ experiments in underground laboratories. Recently, the study of geoenergy has led to a number of in situ experiments, which focus on different aspects of stimulation and circulation [11,12,13,14,15]. In general, in situ experiments help to gain new insights as they provide a novel approach compared with the laboratory or natural scale in terms of scalability, accessibility, and controllability. First, they are closer to the natural full scale than small-scale laboratory experiments. For this reason, in situ experiments can provide insights into the extent to which processes observed in laboratory experiments can be transferred to full scale by bridging the gap. Second, as opposed to full-scale processes that occur at depths where detailed monitoring is not possible and in-depth knowledge of the local boundary conditions, i.e., the geological structures, is missing, in situ experiments allow detailed rock volume characterization and detailed monitoring. This ultimately allows us to study the underlying processes with new precision. Third, a priori knowledge on the test site combined with accessibility to the rock volume via galleries and caverns allows the design of experiments to address specific questions under semi-controlled conditions, while at the same time exploiting natural complexity, e.g., in geological structures, stress fields, and hydrology, without the limited sample sizes of laboratory experiments.

In situ experiments in geosciences are typically performed at a decameter scale. Recently, projects have strived to step up the scale with hectometer-scale experiments in order to come one step closer to full scale [16]. Conducting in situ experiments on a hectometer scale allows the observation of processes even closer to the full scale, and also demonstrates to stakeholders and the public that technologies, e.g., in geothermal energy, can be successfully implemented and controlled. In the Roadmap for Deep Geothermal Energy in Switzerland, which was developed to guide research and development for the Swiss Competence Centre for Energy Research—Supply of Energy (SCCER-SoE), scaled experiments are recognized as a key pillar for geothermal energy research and led to the performance of laboratory experiments and decameter-scale experiments in the Grimsel underground laboratory. Hectometer-scale experiments are now being performed in the newly founded Bedretto Underground Laboratory for Geosciences and Geoenergies (BULGG), which is located in the Furka side tunnel (Bedretto window) below the Swiss Alps.

In this paper, we discuss the technical aspects of realizing high-quality, multi-disciplinary, low-risk monitoring in hectometer-scale experiments focusing on passive seismic monitoring, hydro-geomechanical monitoring, and surveillance using active monitoring methods to screen the rock volume. We start with a comprehensive general discussion on the risks for monitoring that come with such large underground experiments. We demonstrate that risks and set-up are quite different from the more common decameter-scale experiments performed in situ. Second, we introduce as a case study of the recently installed monitoring system of the Bedretto Reservoir Project (BRP), an in situ experiment on a hectometer scale realized in BULGG after extensive technical development.

## 2. Challenges in the Instrumentation of Meso-Scale Experiments

Stepping up the scale of in situ experiments from decameter to hectometer scale is expensive, both in a financial and organizational manner. Moreover, instrumenting these experiments comes with additional challenges which we discuss here.

### 2.1. Borehole Deviation

**Figure 1:**
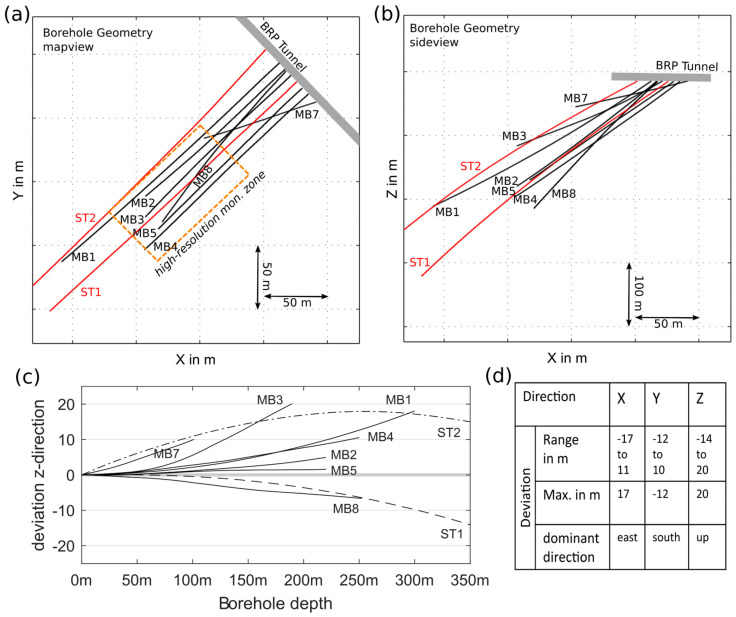
Boreholes of the Bedretto Reservoir Project (BRP) shown in (**a**) map view and (**b**) side view. Monitoring boreholes are shown as black lines; production boreholes are shown as red lines. In (**c**) the sum deviation between the straight boreholes trajectories planned and boreholes realized in the z-direction is shown. Most boreholes deviated upwards at distances >100 m from the borehole mouth. The maximum deviation is 20 m. Horizontal and vertical deviation is summarized in (**d**).

High-resolution monitoring requires placing sensors within an experimental volume of some hundred-meter extension, i.e., in boreholes of some hundred-meter length. Long boreholes, especially in hard rock, have a considerable risk for deviations and seldom reach the same straightness as shorter boreholes (Figure 1) due to the influence of gravity, geological structures, and foliation. This influences the design of the monitoring system because bringing sensors exactly to a specific point of interest is often not possible. Instead, the possible deviation must be taken into account when designing the monitoring borehole geometry. The exact options for sensor positioning are clear only after the boreholes are drilled. Moreover, the deviation in borehole drilling prevents closely spaced boreholes because the intersection of boreholes must be avoided. The design distance necessary between boreholes is individual and depends on the geology, the drilling technique used, and other local conditions such as the available space for the drill rig.

### 2.2. Borehole Roughness

**Figure 2:**
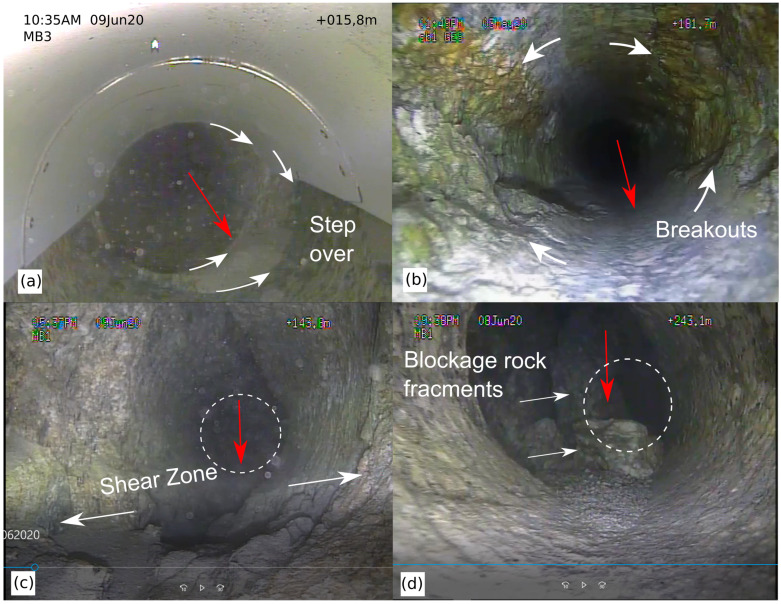
Quality of hectometer-scale boreholes with rough borehole contours: (**a**) step over at end of conductor pipe, (**b**) borehole breakouts, and (**c**) shear zones are common observations and major challenges for lowering monitoring equipment. In addition, (**d**) borehole blockage by rock fractures is not unusual. White arrows highlight features; red arrows point towards the borehole bottom, as the borehole camera is rotated. Pictures taken of monitoring boreholes MB1, MB3, and MB4 of the BRP project. Figure 2b modified from [17].

Higher stresses acting on long boreholes often come with borehole deformation, especially borehole breakouts [17] because the boreholes are located outside of the stress shadow of the underground cavities. In addition, faults or other zones of weakness can cause steps in the borehole direction and reduce the borehole smoothness further. Thus, cored boreholes may not have walls as smooth as short boreholes and exhibit, at least partially, a topography (Figure 2). Borehole roughness as well as loose particles can cause deadlock of the monitoring system during installation before the anticipated borehole depth is reached or cause damage to sensors and cables. This is, therefore, a prominent risk to consider, not only for (sub)horizontal boreholes. Loose particles that can become clamped between the borehole side wall and sensors come from either drilling-related debris or represent loose parts falling from the monitoring system itself. Debris is often present in long boreholes because the complete cleaning of long boreholes is much more difficult and often not possible, especially in boreholes with continuing rock fall.

### 2.3. Multi-Sensor Installation 

**Figure 3:**
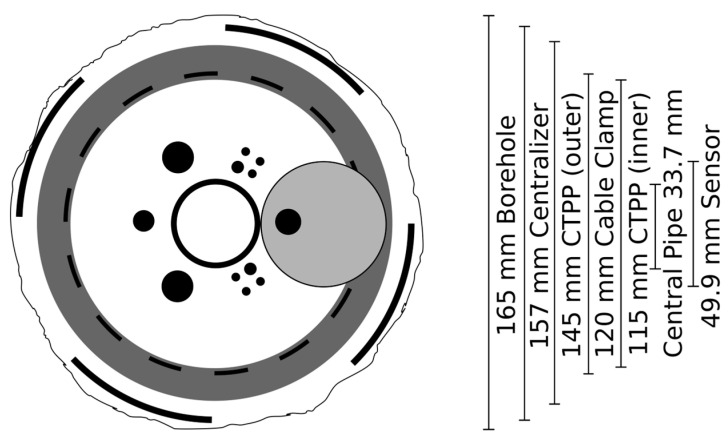
Illustration showing the limited space inside the borehole (crosscut). The sketch shows the geometries of the most dominant devices installed in the BRP project with respect to the borehole diameter (most outside thin black line): the solid lines show the outer diameter of the centralizer springs; the dashed line shows the diameter of the cable clamps; the dark grey ring shows the cementable tube pore pressure; the bright grey circle shows the largest sensor installed next to the central rod (accelerometer); the solid black line in the center shows the central tubing; and the solid black circles show the different cables. All devices are separated in space to improve consistent cementation. Details in text.

For decameter scale in situ experiments, it is a common monitoring approach to have separate monitoring boreholes for different instruments, e.g., a few for seismic monitoring and additional boreholes for hydro-geomechanical monitoring [5,8,18,19]. Long boreholes are expensive and the borehole deviations discussed above discourage closely spaced boreholes. Therefore, a smaller number of boreholes should cover a larger rock volume, which means that more and different kinds of sensors should be accommodated in the same borehole. All cables, sensors, and clamps must be fitted into the limited borehole space and bypass each other (Figure 3). Different sensor systems come with different requirements in how to insert and clamp the sensors best. Finding an installation scheme that suits all sensors, while avoiding negative sensor interference, puts constraints on the sensors installed and on the borehole diameter. Additionally, to avoid the system getting stuck during installation and to avoid damage to sensors and cables, it is necessary to calculate a larger safety margin between the borehole side wall and the instrumentation than in short boreholes. Thus, the usable space inside a hectometer-scale borehole is smaller than in a decameter-scale borehole for boreholes with the same diameter. Note, due to drill bit wearing, the borehole diameter in long boreholes can be a few millimeters smaller in the deep sections. Finally, the risk of loose cables that cause blockages (and damage to the cables) needs to be addressed.

### 2.4. Pressure

Both water and pressure are a significant challenge for many sensors, especially if electronic parts are present. For several reasons, both water and pressure play a more dominant role in hectometer-scale experiments and pose another demanding boundary condition and risk to the experiments. First, due to logistics, long monitoring boreholes in hectometer-scale experiments will often be drilled downwards, which in many geologies means that the borehole will collect mountain water. Hydrostatic pressure in some hundred-meter-long boreholes easily reaches a few Mega Pascal. In addition, hectometer-scale experiments can utilize natural pressure conditions if the monitoring boreholes are closed and do not drain the experimental volume. In these conditions, the monitoring equipment is subject to the natural pore pressure, which depending on the general setting and the overburden rock mass at the experiment site, can easily reach pressures of 10 MPa and higher. This is contrary to decameter-scale experiments, which are placed next to existing cavities where the rock volume is already drained. For completeness, we point out that in addition, the acidity of mountain groundwater can cause problems because it can lead to a significant, fast corrosion of the monitoring equipment. In some installations, it is furthermore necessary to take into account the buoyancy as well as the dragging force introduced onto the equipment by flowing mountain groundwater.

### 2.5. Heavy Equipment

Logistically, larger systems come with more material and more weight, which makes them cumbersome. Even a multi-component monitoring system in a borehole of only 150 m length can easily reach a weight larger than 1000 kg. The risk to people and equipment is increasing with longer boreholes, which means that more safety measures are needed. One example is the clamping system needed for long boreholes, which is normally not necessary in the installation of decameter-scale experiments. For steep boreholes, the fixation system must ensure that uncontrolled sliding of equipment into the borehole is avoided despite the weight. Shallow dipping boreholes require a system that enables actively pushing the system inside because, due to weight and bulkiness, the limits of pushing it by hand are reached early. In both cases, the fixation system must be able to pull the equipment in a controlled manner. In water-filled boreholes, the actual acting weight can be different from the physical weight of the equipment due to buoyancy effects. For heavy systems, pulling forces can elongate cables and rods. The elongation can be different for different components and must be taken into account in the network design.

In addition to the system’s weight, it is also the system’s length that plays a role in the network design. Whereas in decameter scale projects, rod systems are available that can control the orientation of sensors, this is not the case for large boreholes because torque is more severe. Many systems, e.g., ordinary rod systems or drill pipes, are prone to corkscrew torque over the length of some hundred meters, especially if the friction gets too high. For hectometer-scale experiments, this means that the sensor orientation will change in depth when pushing the instrumentation into the borehole, which is a problem for oriented sensors such as seismic sensors.

### 2.6. Borehole Sealing

Hectometer-scale experiments are of special interest to the scientific community because they allow access to natural conditions. Open boreholes introduce stress perturbations that alter the natural stress field, drain the rock volume, and introduce shortcuts for fluids along the wellbore. In order to keep the rock volume undisturbed and under natural conditions, it is necessary to seal the monitoring boreholes. This is carried out most effectively using cement or grout with similar material properties as the overall rock condition to reduce the boreholes’ impacts on the experiment. Even with sealing, monitoring boreholes alter the rock volume, especially if pathways for fluids remain open. In order to reduce the impact, it is necessary to reduce artifacts by proper slurry design. For example, shortcuts in cemented boreholes are likely through a micro-annulus that can form during curing. Other typical pathways for fluids are pathways inside cables, along casing, and in between a bundle of cables. These unwanted pathways can in most cases be suppressed by cement design and other technicalities, e.g., cable clamps as shown below.

On the other hand, sealed boreholes come with their own risks. As discussed above, any closed borehole increases the pressure acting on the monitoring system. Another risk to consider in the experiment design is the sensor–grout interaction: temperature increase during curing, humidity, or very high pH values can affect or damage sensors and cables and must be carefully considered in the cement design. In addition, different sensors have different grout integrity requirements. Whereas some sensors (e.g., acoustic emission sensors) require a full space cementation without openings such as large air bubbles in order to ensure good sensor coupling and the unaffected reception of signals, other sensors require open borehole sections without grout (e.g., pore pressure sensors). The careful design of a sealing concept that is suitable for all sensors is for these reasons mandatory in realizing high-resolution monitoring in hectometer-scale experiments.

## 3. Application to a Deep Underground Geothermal Reservoir Project

### 3.1. Introduction of the Bedretto Reservoir Project (BRP)

In the following, we present the monitoring network of a hectometer scale in situ experiment from the field of geothermal energy. The Bedretto Reservoir Project (BRP) is located in BULGG and conducts studies in in situ techniques and procedures for a safe, efficient, and sustainable use of geothermal heat. It aims at gaining in-depth knowledge on rock response to injection and water circulation at the hectometer scale, knowledge that ultimately aims at controlling risks in geoenergy exploration. BRP uses a multi-component monitoring system combining sensors from seismology, applied geophysics, hydrology, and geomechanics (Table 1) installed inside long boreholes (up to 300 m in length). Boreholes are drilled from the Bedretto tunnel. Boreholes are sealed to reach (as far as possible) rock integrity within the experiment volume. The BRP project is an underground injection experiment at the hectometer scale aiming at creating a geothermal reservoir of more than 100 m diameter for water stimulation and circulation under controlled conditions. The scale and location separates BRP clearly from other recent stimulation experiments such as the FHF experiment at the Äspö Hard Rock Laboratory [12,20,21], the ISC project at the Grimsel Test Site [13,19,22], the Collab-experiment at the Sanford Underground Research Facility SURF [14,23], and the STIMTEC experiment at the Reiche Zeche Freiberg mine [15] as presented in Table 2.

#### 3.1.1. Scientific Background

In enhanced geothermal energy production, water is injected into a hot reservoir where it flows through the hot reservoir and hot water is extracted through production wells in order to make geothermal heat accessible at the Earth’s surface. The exploration of geothermal heat for energy or heat production is of growing interest in the context of developing sustainable energy resources for countries worldwide. Besides geographical constraints—geothermal heat for the generation of power requires appropriate temperature gradients in the crust locally—the technology is facing two fundamental challenges during operation. First, the operation requires a permeable fracture network in between the producing boreholes. Often, it is necessary to engineer this fracture network or enhance the permeability by hydraulic stimulation. The generation and long-term operation of such permeable fracture networks is still poorly understood. Second, hydraulic stimulation and water circulation are accompanied by induced seismic events both in full-scale operations [24,25,26] and in in situ experiments [12,15,23,27,28]. Seismic events can alter the permeability of the reservoir and are—if significant ground shaking is induced—a threat to surface infrastructure. Controlling induced seismicity in such a way that a certain magnitude level is not exceeded is therefore important. While traffic-light systems have been successfully implemented [26], the underlying processes are still poorly understood.

Recent in situ experiments on the decameter scale were able to observe and analyze small-scale processes. For example, events of induced seismicity were shown to be dominantly correlated to the active stimulation phases and outlined the fracture growth, or more precisely, the progressive reactivation of a fracture network in consecutive injection stages [20,28]. Calculated focal mechanisms displayed heterogeneous fault plane orientations in disagreement with the macroscopic orientation of the fracture [20,28]. Seismic events represent critically stressed fractures in optimal orientation within the local stress field [20]. The observed heterogeneity likely reflects structural heterogeneity of the rock mass and the presence of faults at all scales. The influence of local geology is hereby very pronounced as even injection sites being located only meters apart show a significant variation both in seismic activity and b-values [22]. Another factor potentially influencing the seismic activity is the stimulation scheme [12,21]. Hydro-mechanical-coupled processes were successfully observed and can be subdivided into two main groups [19]: (1) At small distances from the injection point the fluid pressure signals are hydraulically controlled and induce complex variable deformations, such as (competitive) fracture opening, shear dislocation, fracture initiation, and stress transfer. (2) At larger distances from the injection point, the fluid pressure perturbations are poro-elastic (that means mechanically controlled) and the mechanical response is mostly compressional. To which extent the observed processes are relevant for full-scale operations in the long term remains open, which is why hectometer-scale experiments are of importance.

Ongoing research questions are addressed in BRP: (1) Which stimulation concepts are appropriate for enhancing the permeability by orders of magnitudes while minimizing induced seismicity? (2) What are the relationships between the stimulation concept, transient hydro-mechanical response, permanent permeability creation, final effective porosity, and induced seismicity? (3) How can the overall stimulation-affected volume and its sub-zones be described in its spatial/temporal evolution? (4) How does the maximum-induced seismic magnitude scale with the injected fluid volume? Which parameters of the injection site or the engineering of the stimulation influence induced seismicity most? (5) What are the final heat-exchanger properties of the reservoir and how can we optimize it? Finally, we aim at the application of an advanced traffic light system. This requires real-time analysis and joint integration of all data, especially seismicity, pressure, and pump rates.

**Table 2:** Comparison of decameter scale and hectometer scale injection experiments. Due to the depth and distance to the nearest opening (tunnel), the BRP experiment is expected to represent more realistic stress conditions, pore pressure flow, and seismic response than the decameter-scale experiments. A detailed discussion is provided in the text.

	Hectometer	Decameter			
	BRP	Äspö	Grimsel	Collab	Stimtec
Rock volume	100 × 300 × 100 m	30 × 30 × 30 m	30 × 30 × 30 m	30 × 30 × 30 m	30 × 40 × 30 m
Depth bgl.	~1000 m	410 m	480 m	1500 m	130 m
Injection in m^3^	up to 100 *	0.01 to 0.03	0.9 to 1.5	0.02 to 0.65	0.02 to 0.06
Frac. extent	<100 m *	<10 m	<20 m	<20 m	<10 m
loc. AE events	10.000 to 100.000 *	0 to 102	13 to 3103	58 to 426	0 to 5775
Distance **	60 m–400 m *	4 to 25 m	15 to 40 m	25 to 35 m	10 to 20 m
Inj. borehole diam.	216 mm	102.5 mm	146 mm	96 mm	76 mm
Reference	This study	[12,20,21]	[13,19,22]	[23,29]	[15]

* Ongoing experiment. Anticipated values. ** Distance from injection point(s) to tunnel.

#### 3.1.2. Test Site

**Figure 4:**
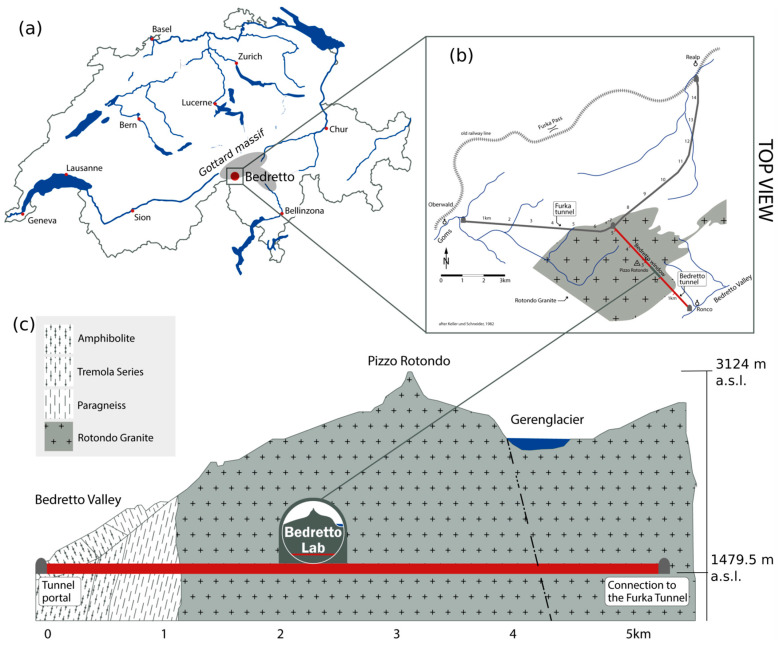
Location and setting of the Bedretto Underground Laboratory for Geosciences and Geoenergies: (**a**) geographical overview of Switzerland; (**b**) map view of local setting with regard to the Furka Tunnel; and (**c**) cross section after Keller and Schneider, 1982. The Bedretto tunnel is shown as a red line.

The Bedretto Reservoir Project (BRP) is realized in the Bedretto Underground Laboratory for Geosciences and Geoenergies (BULGG), which was established by ETH Zurich in 2019. The Laboratory is located inside the 5218 m long Bedretto Tunnel, located below Pizzo Rotondo, the highest peak of the Gotthard massif, a major mountain range in the Swiss Central Alps. The tunnel connects the Furka Base Tunnel with the Bedretto Valley (Figure 4) and comes with 1000 m plus of overburden rock mass, providing a setting that is similar to intermediate depth geothermal reservoirs. The setting provides access to seismogenic depth [16]. BULGG hosts various scales of in situ experiments addressing fundamental questions with, at present, a focus on geoenergy (Bedretto Reservoir Project, BRP) and earthquake nucleation (Bedretto Earthquake Project).

The experiment site of BRP is located at about 2 km from the BULGG tunnel entrance, where a 6 m wide and 100 m long niche was excavated during tunnel construction. The stress environment is dominated by normal and/or strike-slip faulting. The geology within the experiment’s rock volume (approximately 100 m by 300 m by 100 m in size) is composed of weakly deformed Rotondo granite protolith [30] intersected by less frequently distributed highly foliated ductile shear zones and fractures mostly dipping steeper than 50°. The chosen rock volume comes in three distinct lithological units located at approx. 60 m to 120 m, 120 m to 200 m, and beyond 200 m distance (borehole depth) to the tunnel where the Rotondo granite comes with varying degrees of pre-existing deformation, as described in detail in [31]. In the BRP, stimulation experiments are performed in all three units.

The general seismic hazard in the Bedretto region is low to moderate (Swiss Seismic Hazard Model [32]). Only a few microseismic events (M > 2) were recorded in the Gotthard Massif in the vicinity of Bedretto over the last decades including a magnitude 2.3 event of 15 km depth at a distance of 2 km from the tunnel. Microseismic activity observed in the context of excavating the Gotthard base tunnel demonstrates that critically stressed faults exist [33].

### 3.2. Monitoring Network Design

The monitoring network in BRP relies on sensors installed in the tunnel and in monitoring boreholes up to 300 m in length that are instrumented with a large variety of sensors and sealed, as described in detail below. In addition, sensors were installed within the production boreholes. These instruments are focused on monitoring the conditions within the production boreholes correlated to pumping water and pressure build up. In the design of the monitoring concept, it must be acknowledged that resources (especially the number and length of monitoring boreholes) are limited and that high-resolution monitoring of the full rock volume subject to operation is not possible with the resources available. Due to the large dimension of the experiment volume, we used a twofold approach. On the one hand, we realized robust base monitoring throughout the full reservoir; on the other hand, we established a high resolution monitoring zone (length approx. 100 m, radius approx. 40 m) covering the core of the stimulation volume around the production of borehole ST1. Here, we increased the density and spatial distribution of the boreholes and sensors. In addition, we attached great importance to redundancy in the network design in order to acknowledge the technical challenges described above. Sensor failure or, in the worst case, whole borehole loss cannot be excluded but must be expected. Redundancy is achieved in the high-resolution volume by placing more than the minimum number of monitoring boreholes and sensors into the rock volume wherever possible to ensure that monitoring goals are achieved despite sensor failure. In addition, redundancy is considered in the sensor chain design as described in detail below for FBG and seismic sensors.

**Figure 5:**
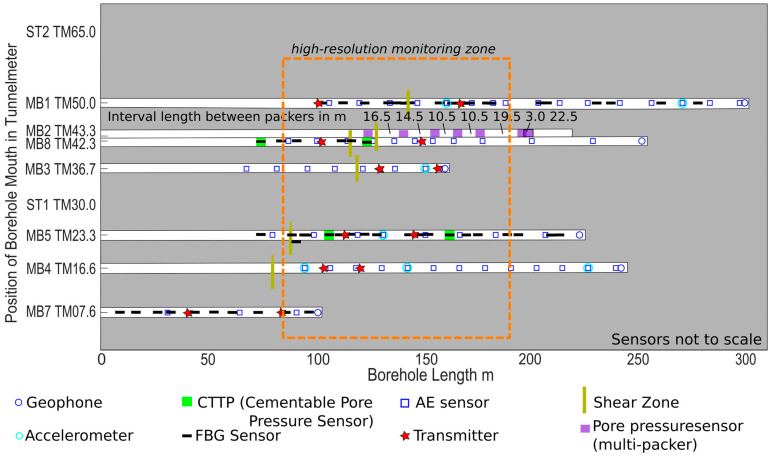
Final sensor positions inside the long monitoring boreholes in the BRP. Monitoring boreholes are sorted regarding the location of the borehole mouth in tunnel meters (TMs). For the borehole geometry see Figure 1.

Designing the sensor geometry requires knowledge on the location of fractures and shear zones. In the BRP, very reliable data were available from the rock characterization, as described in detail in [31] and extensive borehole logging in all monitoring boreholes. Methods used include core analysis, structural mapping, hydraulic characterization, georadar, televiewer, and camera inspection. Combined, we estimated a location certainty for the most important features close to 0.1 m (Shakas et al., in preparation). Sensor positioning relies on the central precision tubing and the sensor chain design as described below. Comparing the theoretical and realized position of sensors relative to the tubing allows us to conclude that the sensor position is precise to <0.1 m. The sensor geometry was designed on theoretical calculations and boundary conditions specific to each sensor type. In general, no sensor position was allowed to coincide with other sensors or the sockets of the central tube, existing every 3 m (Figure 5). Of importance in our network design is the positioning of different sensors in close vicinity to each other, in order to obtain information on different observables in the same rock section. We want to advance process understanding by joint measurements of pore pressure, strain, and seismicity using co-located sensors in order to gain new insights into the full rock response. Decameter-scale experiments show that processes vary over very short distances; therefore, only measurements of co-located sensors allow a robust joint interpretation.

All components of the monitoring network installed inside the long monitoring boreholes had to fulfill environmental and technical boundary conditions: (a) pressure resistant to minimum 10 MPa (conservative evaluation of pore pressure due to 1000 m overburden); (b) suitable for base environments up to PH13 (relevant during cementation, mountain water PH~9.6); and (c) sensor dimensions suitable to fit with the installation and guidance system into 165 mm boreholes (Figure 3). No special temperature requirement was necessary as the natural rock temperature in the BRP reservoir is 20 to 22 °C [30] and temperatures during cementation could be controlled, as discussed below. The network aims for the long-term operation of several years.

### 3.3. Boreholes

#### 3.3.1. Boreholes: Objectives and Requirements

For the reservoir project, a total number of 9 downward-inclined boreholes (length 101 m to 404 m) were drilled off the southwest sidewall of the Bedretto Tunnel (tunnel height 3 m to 4 m, tunnel width 6 m) including 2 producing (injection and extraction) boreholes (ST1 and ST2) and 7 boreholes (MB1 to MB8) for monitoring (Figure 1). Downward orientation of the producing boreholes is necessary to generate water-filled boreholes suitable for stimulation. The longest monitoring borehole MB1 was planned to be oriented parallel to the main injection borehole, ST1, at a distance of 20 m. In practice, both the monitoring borehole, MB1, and the production borehole, ST1, deviated as shown in Figure 1, which altered the geometry. Monitoring borehole MB2 crosses the high-resolution zone but dips steeper than ST1. It provides access to the main rock volume for pore pressure monitoring using a multi-packer but is outside the rock volume reserved for pathways connecting the two producing boreholes. Monitoring boreholes MB3 to MB7 realize distances from 6.7 m to 45.4 m from the producing borehole, ST1. These boreholes allow the distribution of sensors throughout the rock volume with a focus on the high-resolution zone. As these boreholes were later fully sealed, they also penetrate the rock volume in between the producing boreholes. Monitoring borehole MB8 is oriented mostly parallel to the dominant shear zone in the experiment volume in order to allow the monitoring of the hydro-mechanical transition zone and the far field outside of the primary injection volume.

#### 3.3.2. Boreholes: Implementation

We decided to drill the boreholes in three batches separated in time. This allows rock characterization in the early project stage while leaving flexibility in design to take into account the outcome of characterization, drilling (borehole deviations), and pre-experiments later on. This increases the controllability of sensor placement.

In the first phase (September 2019), monitoring boreholes MB1, MB2, and MB3 were drilled. The first three boreholes drilled were used for extensive characterization of the rock volume [31]. They span a tripod in such a way that they penetrate potential fractures across the stimulation volume. Furthermore, they provide suitable distances for imaging and characterization techniques, i.e., georadar, cross-hole seismics, and hydrological measurements. The boreholes were diamond drilled for core extraction and only later reamed towards the full borehole diameter. In the second phase (May and June 2020), the production boreholes ST1 and ST2 and MB4 were drilled. Afterwards, monitoring boreholes MB1, MB2, MB3, and MB4 were instrumented and first experiments were conducted. Finally, in spring 2021, the remaining monitoring boreholes were percussion drilled to complete the monitoring system. The orientations of the later boreholes were chosen based on the outcome of boreholes drilled previously and the monitoring results of the first stimulation experiments. In this way, borehole deviations that resulted in sensor re-positioning in space could be taken into account. Due to the limited space in the tunnel, excavations at the tunnel base and in the tunnel roof were necessary to provide sufficient space for the drill rig. For safety measures, to sustain the pressures acting during drilling and to avoid hydraulic shortcuts towards the tunnel, each borehole was cased with a 15 m cemented conductor pipe at the borehole mouth (inner diameter 174 mm). The stimulation borehole ST1 is zonally isolated with a 14-packer system, especially designed to operate under high pressures needed for stimulation.

#### 3.3.3. Boreholes: Results

All boreholes were successfully drilled (Table 3) but differ in length and orientation from the anticipated geometry due to difficulties encountered. The drilled borehole trajectories deviated up to 25 m from the anticipated location, as shown in Figure 1. Hydraulic characterization revealed one major short-cut in between boreholes, namely, a short-cut connecting production boreholes ST2 and MB1. In MB3, a large amount of fine-grained debris entered from a shear zone into the borehole shortly after reaming. About 10 m of the borehole remained clogged, even after extensive flushing. In MB4, large rocks blocked the deepest 2.5 m of the borehole (Figure 2d). Breakouts and step-overs in the dominant fault zones were more severe than expected (Figure 2c).

### 3.4. Installation and Guidance System

#### 3.4.1. Installation and Guidance System: Objectives and Requirements

Three scenarios must be addressed in the design of the installation and guidance system that would result in data loss: (1) cables getting cut during installation due to torque or cables being damaged by squeezing; (2) the whole system getting locked; and (3) the system falling uncontrolled into the borehole or, vice versa, is unable to slide into the borehole because the friction is too high. A guidance and installation system is needed that addresses these risks but allows at the same time enough room for all sensors and for consistent cementation. Using a casing was not possible for BRP because casing creates an additional layer in between the sensors and the rock. This is disadvantageous because (a) these two additional contact planes are prone to develop an annulus opening and compromise the leakage integrity, and (b) the contact plane can dampen or alter signals, e.g., high-frequency (kHz) seismic signals or strain measurements that require direct contact with newly initiated cracks.

#### 3.4.2. Installation and Guidance System: Implementation

In BRP, we developed an installation and guidance system that is based on a centralized tube (outer diameter 33.7 mm) used as cementation pipe and placed in the center of the borehole by centralizers with integrated cable clamps. Advantages of the system include that this set-up enables (a) a gradual, dense cementation; (b) surpassing areas of heavy damage within the borehole; (c) protecting cables and sensors; and (d) a high amount of flexibility in sensor placement, while keeping material requirements and, therefore, costs to a minimum. The limited space for sensors next to the central tube is disadvantageous. The inner diameter of the tube (25.4 mm ID) is just large enough to result in acceptable pressure losses when pumping cement slurries. The precision tubing comes with 3 m long pipes with a guaranteed length uncertainty below 2 mm.

**Figure 6:**
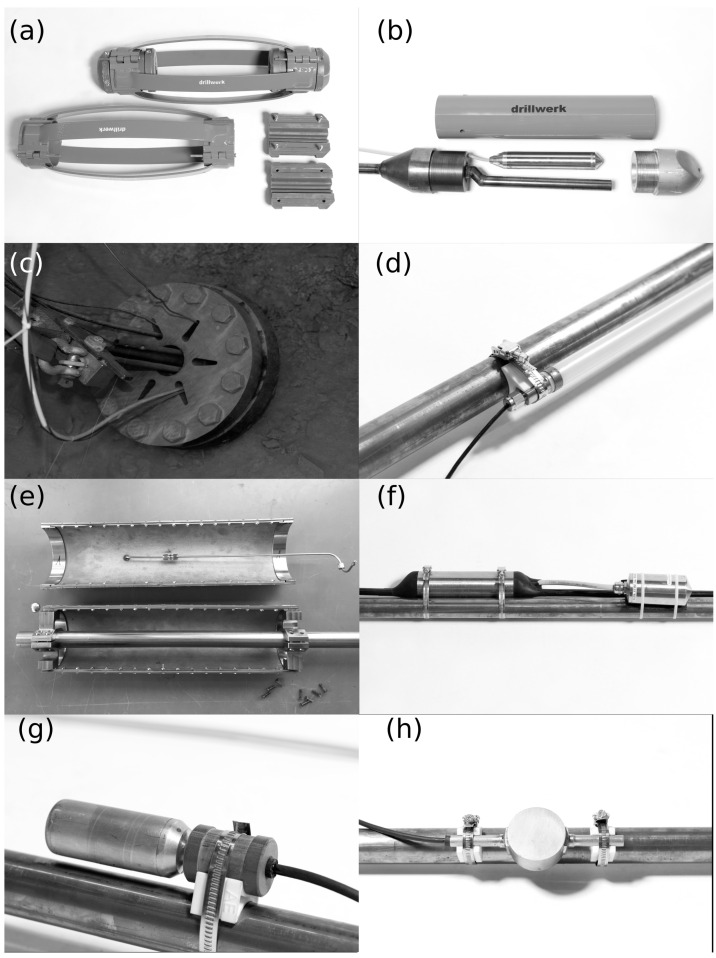
Main components of the BRP multi-sensor monitoring network installed together in the same monitoring boreholes: (**a**) centralizer with integrated cable clamp; (**b**) frontshoe with integrated geophone; (**c**) top plug; (**d**) fiber Bragg grating (FBG) sensor; (**e**) cementable tube pore pressure (CTPP) sensor; (**f**) high-frequency accelerometer with pre-amplifier; (**g**) in situ acoustic emission (AE) sensor; and (**h**) ultrasonic transmitter. For details, see Section 3.4 Installation and Guidance System for (**a**–**c**); Section 3.5 Geomechanics for (**d**,**e**); Section 3.6 Seismology for (**b**,**f**,**g**); and Section 3.7 Active Seismics for (**h**). An overview of size, numbers, and locations is given in Table 1.

**Figure 7:**
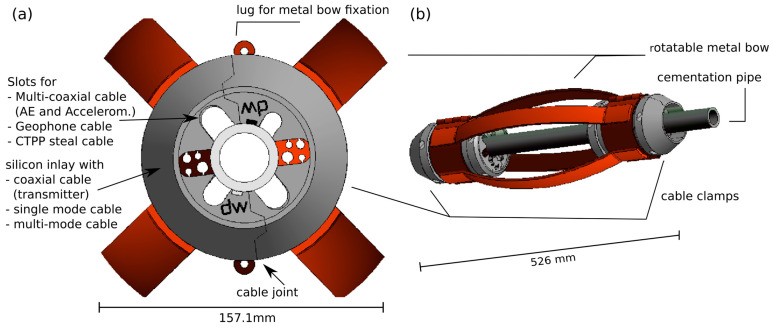
Centralizer with integrated cable clamp: (**a**) crosscut and (**b**) side view. Silicon inlays allow the installation of thin cables.

Centralizers (Figure 6a) were placed approximately every three meters or where increased load by sensors was expected. Whereas traditional centralizers only serve for positioning, we developed a new type of centralizer that addresses many of the risks discussed above. Our centralizer named “practiCABLE” manufactured by Drillwerk, Hanover, Germany keeps the tube centralized and works as a guide and spacer. In addition, it serves as a cable holder, which keeps all cables tight, secured, and separated (Figure 7), even in fracture zones and tight spots where high torsional or radial forces on the tube string can occur. The centralizers support consistent cementation by separating the cables in space and modeling the flow path using the centralizer’s openings to ensure homogenous migration. The cable holder was custom-made specifically for the number of cables and cable diameters used in the BRP.

The installation and guidance system was complemented with closing pieces at the front and the bottom. The guide shoe (Figure 6b) consists of a steel tube serving as housing for the geophone and an aluminum front nose. It comes with considerable weight of 60 kg and serves as a pulling force during the installation in the inclined boreholes. The convex, flattened nose enables improved travel in rough borehole sections. One central and three side-view holes serve as exit points for the classical bottom to top cementation.

At the borehole mouth the system was screwed to the conductor pipe (Figure 6c). The construction serves as an anchor and prevents blow-out in case of pressure build-up, e.g., during cementation. Two different approaches to finalize the clamping at the borehole mouth were used in the BRP: During the first installation phase, a significant amount of pore pressure was expected in the monitoring boreholes, which would require cementation at pressures on a similar level. For this reason, the installation and guidance system was anchored in the first 3 boreholes with a packer suitable for pressures up to 10 MPa installed inside the conductor pipe and a front plate screwed to the conductor pipe. The axial load on the packer is transferred to the top flange. The packer was equipped with a self-swelling cable sleeve around the packer and custom-made for the cables installed in BRP to allow cable feed-through and sealing. The packer is not recoverable after cementation. In the second installation phase, the requirements for the fixation were lowered because measurements showed much smaller pressures than expected. Here, the guidance and installation system was screwed directly to the conductor pipe for monitoring boreholes MB5, MB7, and MB8.

#### 3.4.3. Installation and Guidance System: Results

The installation and guidance system worked well during installation and is seen as one of the key elements that enabled us to carry out the installation. Despite the large number of breakouts, the system did not get locked, not even temporarily. Considering that during borehole imaging we experienced severe problems with logging tools (including lockage of borehole tools, the loss of probes due to cut cables, and probes that could not be lowered to the borehole end), we conclude that the development of the installation and guidance system to reduce such risks for the monitoring system were sufficient and necessary. The system was handy and could be installed without heavy lifting in the limited space in front of the borehole. The weight was fully held by rock anchors attached to the ceiling.

### 3.5. Geomechanics

#### 3.5.1. Geomechanics: Objectives and Requirements

We aim at monitoring the geo-mechanical rock response within the reservoir and in the far field over time. Data should allow for deformation mode analysis from strain measurements. It is, therefore, required to place sensors for strain and pressure monitoring inside the fracture network at dominant features. We also aim at investigating the influence of temperature on the overall performance.

In order to monitor induced transient and permanent deformations (strain) continuously in space and time, different sensor types are necessary. Fiber Bragg grating (FBG, Figure 6d) sensors allow the continuous measurement of strain in time with high frequency. FBG measurements are based on the reflection of laser pulses at artificially induced grid modifications (so-called gratings) along the fiber, reflecting one specific wavelength from the incident wavelength spectrum each, while the rest of the spectrum is transmitted unaffected. We consider FBG sensors as a centerpiece in the network design because they allow us to have a direct physical measurement of the fractures closing and opening. Fiber-optic cables that were installed both for strain and temperature monitoring allow measurements continuously along the whole borehole length, but the distributed measurement comes with time gaps. Hereby, single-mode cables were chosen suitable for distributed strain sensing (DSS) using Brillouin scattering or distributed acoustic sensing (DAS) using Rayleigh Scattering and multi-mode cables for temperature monitoring. The ST1 borehole is equipped with a hybrid cable with a multi-mode loop for DTS and two single-ended single-mode fibers for DAS. The fiber for temperature measurements is combined with a heating line in hybrid mode to allow for active heating of the fiber for detecting the major flow zones within the borehole.

Pore pressure monitoring is normally performed in open boreholes. Cemented pore pressure sensors unfortunately measure the pore pressure signal with a significant delay, thus dynamic and instantaneous pressure changes are not recorded. We, therefore, installed pore pressure sensors in an open borehole with zonal isolation using a multi-packer (Solexperts AG, Mönchaltorf, Switzerland). Boreholes with open borehole sections perturbate the local stress field more than cemented boreholes. We accept the risk that fractures generated during the stimulation might be locally influenced by the open borehole sections and could connect neighboring fractures, although this can alter the fluid migration during stimulation. We consider the risk to influence the experiment significantly as very low because the multi-packer borehole is placed on the edge of the experiment volume. Each interval in ST1 has a downhole pressure sensor with an accuracy of 0.05% of full scale (0.02 MPa) with a maximum pressure of 40 MPa that can be used for monitoring stimulation or static fluid pressure.

In addition to pore pressure monitoring in the open borehole, we are interested in measuring pressure at the same location where we measure strain, i.e., co-locating pressure sensors with FBG sensors in order to simultaneously measure the temporal evolution of strain and pore pressure of a specific fracture. The problem is that FBG sensors must be cemented. We, therefore, developed a new device, the cementable tube pore pressure (CTPP) sensor. The device allows us to measure pore pressure in cemented boreholes at the position of FBG sensors by creating an artificial opening in the cement. The design of the artificial opening must fulfill the following boundary conditions: The open section should be as closely connected to the borehole wall as possible and may not be cemented. Furthermore, it is necessary to install a pore pressure sensor and FBG sensor together within the limited borehole diameter. Strain and pressure are measured only locally at the sensor (point sensor), i.e., sensors must be positioned at representative fractures throughout the experimental volume. The probe is manufactured by Solexperts AG, Mönchaltorf, Switzerland.

#### 3.5.2. Geomechanics: Implementation

For strain monitoring, we installed FBG sensors os3600 by Micron Optics Inc. (Figure 6d). The 1 m long sensors were placed across single or multiple fractures and shear zones within the boreholes. The sensors were positioned for base monitoring at dominant features only. In the high-resolution zone, FBG sensors are placed more densely on first- and second-order fracture sets. FBG sensors can record strains in borehole direction with a sample rate of 1000 Hz at a resolution of +/−1 µε with an accuracy of 0.85 µε. By down sampling the data to 1 Hz with a forward moving average [34], the resolution is increased to 0.1 µε. FBG sensors were attached to the central pipe of the installation and guidance system using 3D-printed brackets. For laser pulse transmission and data recording, we use the Micron Optics Inc. (Atlanta, GA, USA) si255 interrogator. Seventy sensors were installed in four boreholes (MB1, 5, 7 and 8, Table 2). The sensors were arranged in series along two chains per borehole (except in MB7 which has only one chain) with ten sensors in each chain. Ten sensors per chain ensures that the specific wavelength of each grating is clearly separated from other FBG sensors. Sensors were positioned in alternating order in the two chains. In this way, the loss of one cable, e.g., due to damage during installation, does not lead to data loss in a wide section of the borehole. Moreover, for redundancy, the single-mode fiber-optic cable connecting the FBG was installed inside a loop, which allows access to the gratings from both sides. We placed the sensors at fractures with a high likelihood of being open and conductive based on observations in the geophysical logging [31]. FBG sensors were cemented into the borehole in order to ensure proper coupling to the surrounding rock volume.

Fiber-optic (FO) cables were installed both for strain and temperature monitoring in all monitoring boreholes but MB2. The cables run next to the other sensors, which allows the direct comparison of data and the comparison of different instrument types. For example, the strain measurements of FBG sensors and FO cables will be compared in future experiments as well as the seismic recordings of AE sensors and fiber-optic-based DAS systems. We used two different single-mode cables for strain monitoring manufactured by Solifoss: the BRUsens V9 grip cable (loop), which comes with one tight-buffered optical single-mode fiber, and the BRUsens DSTAS V13, which comes with two tubes with one tight-buffered optical single-mode fiber each (strain range up to 10,000 µε). The BRUsens DSTAS V13 cable contains one additional gel-filled stainless steel tube for loose buffer. This tube contains one multi-mode fiber loop for temperature measurements and four single-mode fibers. Two of the loose single-mode fibers are connected to one tight-buffered single-mode fiber each to create two identical loops. Both cables come with a structured polyamide outer sheath for improved mechanical cement coupling and metal tubes around the fibers for shielding. We used BRUsens DSS V9 because of its smaller diameter (3.2 mm) during the first installation phase to allow more room for the cementation and switched to BRUsens DSTAS V13 (diameter 6.5 mm) after the successful first installation because it allows more simultaneous measurements. All fiber-optic cables were fixed to the installation system using the cable clamps of the centralizer as described above. We used the Dual Vision interrogator manufactured by Omnisens for distributed strain (DSS) measurements with a spatial resolution of 1 m and an actual accuracy of 5 to 10 µε using the BOTDA (Brillouin Optical time domain analysis) method. For temperature measurement, a Silixa XT interrogator was used with a temperature resolution of 0.01 °C and a spatial resolution of 0.254 m with a measurement time of 1 min per channel. Different interrogators will be used throughout the experiment. Fiber-optic cables are suitable to record microseismic events using distributed acoustic sensing (DAS), but for picoseismic observations (seismic events with magnitudes M < <−2) the gauge length of the cables is a severe limitation. Because potentials and limitations of DAS for seismic monitoring in in situ experiments are partially unclear, we will use the BRP network for benchmark measurements.

For pore pressure monitoring, we used a classical multi-packer installation in MB2 with pore pressure sensors positioned at the wellhead locations and hydraulically connected to downhole intervals. Our multi-packer comes with seven 1-meter long packers (outer diameter 88 mm) in order to isolate intervals with lengths ranging from 1.5 m to 22 m. The intervals were chosen based on the fracture/fault clusters as identified from the core analysis and logging constrained by the borehole wall quality for packer placement. The installation depths of the packers are shown in Figure 5. For pressure monitoring, we used the highly precise Pressure Transmitter Series 33X by Keller (Winterthur, CH, Switzerland). The floating piezo-resistive transducer is able to detect pressure variations with an accuracy of 0.05% of full scale (0.0075 MPa) with a maximum pressure of 15 MPa. Data are digitized using the Geomonitor system. We placed the multi-packer system in borehole MB2, located outside of the direct connection path between the production boreholes to reduce the impact of the open borehole on the stress field and fluid flow. The multipacker-system provides zonal isolation. The locations of the packed intervals are designed based on available information, such as from geological structures interpreted from well logs and drilled cores, hydrotests, and GPR in order to minimize the cross flow between different intervals. All individual intervals are accessed hydraulically via separate hydraulic lines, which are normally closed at the surface to avoid drainage of the reservoir volume. 

**Figure 8:**
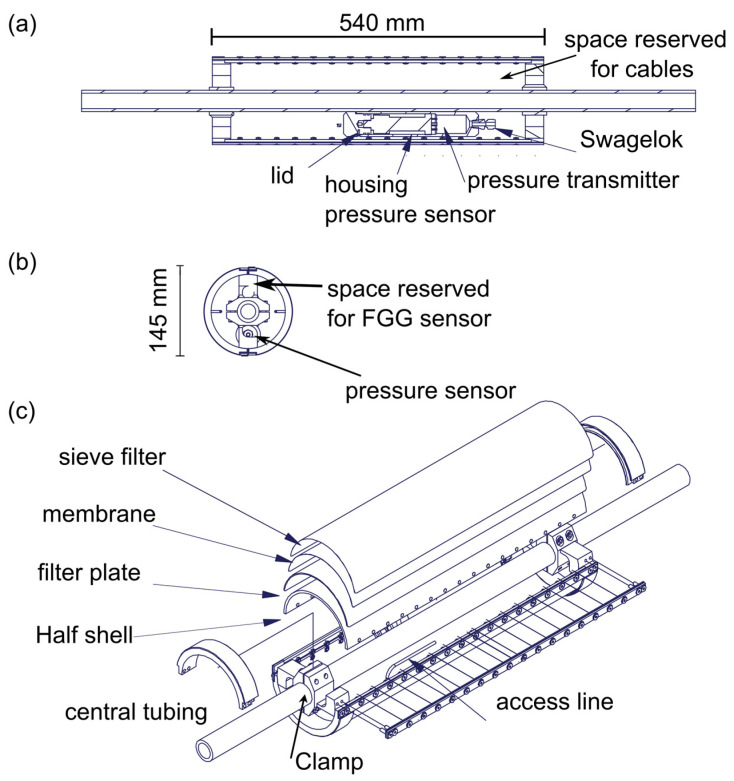
Composition of the cementable tube pore pressure sensor (CTPP). Shown is (**a**) crosscut along tubing; (**b**) crosscut orthogonal to tubeing, and (**c**) disjointed components. The sensor was developed to create an open space within the cemented borehole to allow the measurement of dynamic pressure signals. The construction ensures that all cables of other devices can pass through the interior while also installing an FBG sensor at the same location.

In order to fulfill the requirements for true pore pressure monitoring in cemented boreholes (see Section 3.4.3), we developed the Cementable Tube Pore Pressure Sensor (CTPP, Figure 6e). We installed four CTPP sensors during the second installation phase in two monitoring boreholes (MB5, high-resolution monitoring zone and MB8, base monitoring) as shown in Figure 5 and focused our observation on two major fault zones. The interior hollow part of the tube (inner diameter of 115 mm), which also hosts the installation and guidance system, all cables, FBG sensor and pore pressure sensor, is fully cemented when sealing the monitoring borehole. The tube’s outer shells, on the other hand, create an uncemented ring volume close to the borehole wall. The latter is realized with multiple shell-type layers: (1) a steel mesh to protect the inside of the shells from the borehole wall while letting the fluid go into the device; (2) a membrane layer which only allows water to pass through but no cement slurry; (3) a sintered filter; and (4) a carrier half shell (Figure 8). The designed system prevents the cement slurry from entering the inner shell during the cementation operation, while maintaining the hydraulic integrity between the implemented pressure sensor and the outer rock volume after the cement is set. Hereby we assume that the narrow opening (maximum of 14 mm) between the borehole side wall and tube will end up with a poor cement quality due to a much lower cement flow resistance in the center. In this way, direct water channels from rock to shell, where pressure is measured, are maintained. The device comes with an outer diameter of 145 mm, which is the largest diameter we dare to bring into the monitoring borehole. The shell is implemented as two half-shells to allow installation without a pulling cable nor a central tube through the device. Fluid pressure is measured using digital piezo-resistive pressure transmitters by Keller (Series PA-23SX) that are attached to the carrier tube (the innermost layer of the CTPP, which is attached to the central tubing of the guidance and installation system using brackets). The sensors measure a maximum pressure of 30 MPa with an accuracy of 0.015 MPa. The data cable of the sensor passes through a stainless steel cable all the way to the surface to prevent the intrusion of water into the electrical connection part of the sensor. The communication with the digital sensors is realized via the BUS system which enables connecting multiple CTPP sensors via one data cable per borehole. 

#### 3.5.3. Geomechanics: Results

Data are successfully measured [35]. All FBG sensors were installed at their designated position; however, eight FBG sensors were lost because the fiber-optic cables were damaged during the installation by accidental mishandling. Luckily, owing to the redundant set-up, most FBG sensors, even those in the damaged sensor chain, are still accessible from the other cable side. Fourteen FBG sensors installed inside MB1 had a distorted signal in May 2022 of unknown origin but have worked fine since then. All FO cables recorded data. 

**Figure 9:**
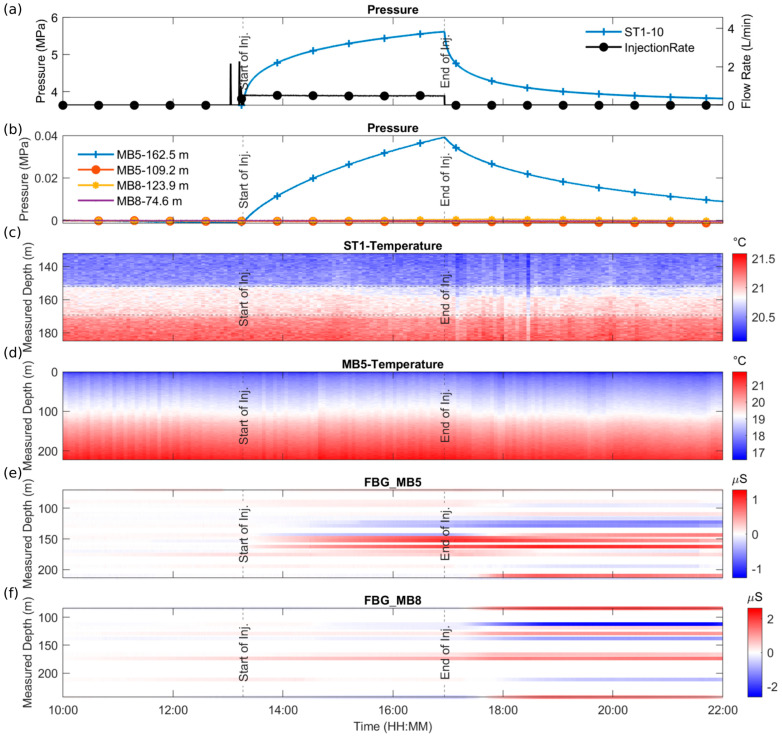
Data examples for BRP sensors (geomechanics). The thermo-hydromechanical monitoring of the reservoir volume during an injection operation in Interval-10 in the ST1 wellbore is shown: (**a**) injection pressure (measured downhole) and flow rate; (**b**) pressure change measured at four CTPP sensors in MB5 and MB8 wellbores, all zeroed at the first data point in the plot for comparison purpose; (**c**) temperature profile measured using DTS in injection wellbore ST1 (the packed injection interval is bounded with dotted lines); (**d**) temperature profile measured using DTS in monitoring borehole MB5; and (**e**,**f**) strain measurements in two wellbores, MB5 and MB8, using FBG sensors with compression shown as negative values, smoothed over 20 s time window. The start and end of the injection are marked in all subplots. The grouted CTPP pressure sensor MB5-162.5 m and the corresponding collocated FBG sensor at the depth of 162.5 m in MB5 clearly show the hydromechanical connection to the injection interval (Interval 10) in the ST1 wellbore.

The four CTPP sensors were installed at their designated position without problems despite their large diameter. Pore pressure measurements show fast and dynamic response similar to pore pressure measurements in the open borehole MB2; therefore, we are optimistic that the creation of free flow channels from the fractures to the sensor’s membrane worked out. Example data of FBG sensors, FO cables, and CTPP sensors are shown in Figure 9.

### 3.6. Seismology

#### 3.6.1. Seismology: Objectives and Requirements 

In BRP, the recording of seismic signals over a significant magnitude range (−5 < M < 2) is necessary. On the upper end, we aim at recording microseismic events (M > 0, frequencies f < 200 Hz) occurring naturally in the wider surrounding of the Bedretto tunnel in the Gottard massif and induced microseismic events (M~0.5) as expected during the stimulation experiment, if 100 m^3^ water are injected [35]. Note that at short receiver distances, microseismic events induce significant ground shaking and can cause damage to infrastructure and the tunnel. For example, a seismic event M~1 occurring within 30 m of the tunnel can reach peak ground velocities (PGV) of up to 200 mm/s. Damage to the tunnel is expected for PGV > 60 mm/s according to the Bedretto risk study [36]. On the other side, even small fluctuations are expected to play an important role and provide qualitative information in the overall system. We, therefore, aim at the recording of induced seismic events down to M-5 (picoseismicity), which means that frequencies up to 50,000 Hz must be covered [10]. Furthermore, we aim at the recording of low-frequency signals observed in previous in situ stimulation experiments [12,15] that are speculated to represent aseismic, pressure-driven deformation. The quality and quantity of the recorded seismic data depend on the sensitivity and the frequency bandwidth of the monitoring network. Data analysis on the seismic index, the energy budget, source mode (moment tensor analysis), and fracture geometry are anticipated in order to compare the seismicity of different injection intervals and different stimulation schemes. Data should allow for the analysis of the temporal–spatial evolution of the stress field using focal mechanism inversion. In order to record seismic signals up to 100 kHz, we aim at source–receiver distances of approximately 20 m to 50 m. Different from hydro-geomechanical observations, seismic sensors record not only seismic activity at the sensor’s position but also record signals from larger distances, in this way providing information about the whole rock volume of the experiment.

#### 3.6.2. Seismology: Implementation

Because no seismic monitoring system covers the recording of this wide range of seismic signals, three different seismic systems were implemented using (a) strong motion/broadband seismometers; (b) geophones; and (c) high-frequency accelerometers and AE sensors (Table 4). For the recording of low-frequency signals (0.01 Hz < f < 125 Hz) and high amplitudes, we installed four strong motion stations (Episensor by Kinematics) that were placed along the tunnel and one broadband sensor (STS-2, Streckeisen, co-located with the Episensor). These sensors were installed on bed plates on the tunnel floor and protected with metal barrels that were screwed tightly to the ground. Data are streamed using Nanometrics Centaur digitizers at sampling rates of 250 Hz and integrated into the Swiss National Seismic Network, which comes with additional surface stations in the Bedretto region [37].

For recording of seismic events with frequencies f < 600 Hz (magnitude range −2.5 < M < 1), we installed 8 triaxial geophones within the BRP volume. Geophones allow the unsaturated recording of waveforms (i.e., without clipping) in this magnitude range even from short source–receiver distances of a few tens of meters. Higher frequencies up to 1000 Hz are recorded as well but come with spurious frequencies. At the bottom of each cemented monitoring borehole (effective borehole length between 96.8 m and 299.5 m), we installed triaxial 100 Hz geophones (Figure 6b). We did not find a geophone suitable for the BRP objectives (pressure resistance, small diameter, and suitable for cementation), especially because many manufacturers could not provide information on the absolute instrument response summarizing both the sensor and the housing. For the borehole installation, we therefore asked the Institute of Mine Seismology (IMS), Somerset West/South Africa, to build a custom-made geophone using 3 uniaxial GS-100 sensors from Geospace (natural frequency 100 Hz), which were tested in our department [38], and to combine it with the IMS geophone borehole housing for high pressures (<20 MPa) typically used for the IMS 14 Hz geophone. The GS-100 sensor is spurious free up to 600 Hz. The instrument response of the geophone (sensor plus housing) is at the moment unknown and will be investigated in future calibration experiments. For the moment, we assume that the housing has a neglectable influence and use the sensor instrument response curve. Nonetheless, the IMS housing has been tested with mining-induced seismicity (personal communication with Dolf Bredenkamp IMS), i.e., with seismic signals whose frequency content and amplitudes are comparable to what we expected to encounter in BRP, which reduces the risk of observed unfavorable resonances. The geophone’s outer diameter is 56 mm; therefore, the sensor did not fit in the open space next to the central tubing. To realize the sensor installation, we incorporated the instrument into the front shoe, where a slight detour of the cementation pipe can be realized (Figure 6b). The sensor is cemented into the front shoe before installation in order to ensure optimum coupling. Then, 2 additional 100 Hz geophones MBAS-3C by Geotomographie were installed in shallow 5 m long open boreholes along the tunnel axis in order to improve the spherical coverage. These geophones are built with three RTC-100 Hz sensors in a triaxial arrangement inside and come with a pneumatic clamping mechanism for simple borehole installation. In addition, one Avalon analog geophone PSS is installed in injection borehole ST1 at a 386.6 m depth. This geophone comes with four HGS sensors (uniaxial 15 Hz geophones) per axis.

For the recording of seismic signals with frequencies above 600 Hz, we installed high-frequency accelerometers and highly sensitive in situ acoustic emission (AE) sensors. Accelerometers were placed in four AE sensors in six monitoring boreholes (Table 1), optimizing the network coverage and sensitivity. We installed seven triaxial accelerometers manufactured by IMS in the BRP borehole network (Figure 6f): four sensors of type ACC-3A25 (IMS, Kingston, Australia) contain three uniaxial sensors of type Wilcoxon 736 (Wilcoxon Sensing Technologies, Frederick, MD, USA); three accelerometers of type ACC-45A19 (IMS, Kingston, Australia) have one triaxial sensor Endevco 45A19 (Endevco, Halifax, VA, USA) inside. The Wilcoxon 736 sensor was at the time of installation the accelerometer with the highest maximum frequency (25 kHz +/−3 dB) on the market. Sensor Wilcoxon 736 comes with a sensitivity of 100 mV/g and an internal noise of 150 μg/√Hz. The Endevco 45A19 has a lower maximum frequency of 7.5 kHz (+/−1 dB) for the y- and z-components and 5.5 kHz for the x-component, but it is more sensitive than the Wilcoxon 736, with 1000 mV/g and an internal noise < 50 μg/√Hz. The accelerometer housing is based on the housing of sensor IMS-ACC25 (IMS, Kingston, Australia) that also incorporates 3 Wilcoxon 736 sensors and can withstand pressures up to 20 MPa. The housing was modified for BRP to remove 2 spurious frequencies at 3 kHz and 10 kHz (a frequency range crucial for the BRP experiments) that were identified during sensor evaluation. In the new design, the longest axis of the housing was shortened and the internal mounting mechanism of the individual sensor elements was modified in order to shift the resonances to higher frequencies. The new housing was tested in the laboratory for pressures up to 10 MPa. Similar to the geophone, a full instrument calibration still remains to be conducted because so far no shake table is available to handle the significant weight of 1 kg and frequencies of up to 25 kHz. During the BRP installation, accelerometers were attached directly to the central tube using cable strips with a neoprene sheet for de-coupling between the sensor and the central tube.

The smallest events expected in the BRP experiment are both in absolute energy and frequency range outside of the recording capabilities of geophones and accelerometers and can be recorded with AE sensors only. AE sensors are not pendulum-based sensors, but exploit the piezoelectric effect. AE sensors do not have, by definition, a flat instrument response, as they come with resonance frequencies which increase the sensitivity. Like pendulum-based seismometers, the sensors can be designed for a wide range of settings, differing both in sensitivity, bandwidth, and the amount of resonance they display. The instrument response is mostly unknown. A comprehensive overview about in situ AE monitoring in decameter-scale experiments is given in [39]. For in situ AE monitoring in BRP, we used the custom-made GMuG-Ma-Blc-30-35 sensor from GMuG, Bad Nauheim, Germany (Figure 6g). The dominant resonance of the sensor is 35 kHz, with a main frequency bandwidth from 1 kHz to 50 kHz, i.e., in agreement with the dominant frequency range of −5 < M < −2 seismic events. The bandwidth is a bit smaller than in previous decameter-scale experiments [12,15,22] since reducing the bandwidth enables increasing the sensitivity [40]. The sensors are embedded in a brass housing suitable for cementation and pressures up to 10 MPa. The sensor has a cylindrical shape, i.e., the sensor has no directivity orthogonal to the borehole orientation. AE sensors are clamped to the central rod using 3D-printed holders, brackets, and Rovatex cylinders for acoustic decoupling.

In order to reduce the number of free cables inside the monitoring boreholes, we developed the picoseismic sensor chain containing both accelerometers and AE sensors. The sensor positions along the chain were custom made taking into account a sensor geometry that allows good spherical coverage of the rock volume and placement preferable in rather unfractured borehole sections. All accelerometers were placed 10 cm from the AE sensors to allow for sensor calibration [41,42]. The picoseismic sensor chain is the backbone for seismic monitoring in BRP, covering the recording of most seismic events in the frequency range from 50 Hz to 50,000 Hz, which corresponds to a magnitude range of approx. −5 to 0. The sensor chain is based on a custom-made multi-coaxial cable (diameter 12.7 mm). It accommodates eleven coaxial cables of type RG178. They were bundled around a central strength member and shielded by a tinned copper braid as well as a polyurethane outer sheath. To avoid water flow inside the cable, the inner bundle is sealed with a silicone compound every 1 m and the outer jacket comes with a swelling, water-blocking tape. Incorporating sensor chains with one multi-coaxial cable rather than using one single coaxial cable for each sensor component reduces the risk of water flow along cables. A maximum of two sensor chains per monitoring borehole were installed.

For the operation of both AE sensors and accelerometers, preamplifiers are needed and incorporated into the sensor chain. The development of a robust and pressure-resistant housing for the electronics of the preamplifier was a severe challenge. After several prototype developments, we developed preamplifiers with each of the cable split-offs and electronics packaged into a steel tube under atmospheric pressure and grouted with resin. Preamplifiers were clamped to the central rod using hose clamps. For the AE sensors, 30 dB preamplifiers were chosen; the for accelerometers −10 dB preamplifiers were selected. Data are recorded continuously with 200 kHz sampling frequency using a 128-channel data acquisition system (GMuG-AEsystem by GMuG, Bad Nauheim, Germany). In addition, 32 channels are recorded in trigger mode with 1 MHz sampling frequency using a 32-channel GMuG-AEsystem with GMuG trigger-mode analysis software (Version Bedretto).

#### 3.6.3. Seismology: Results

**Figure 10:**
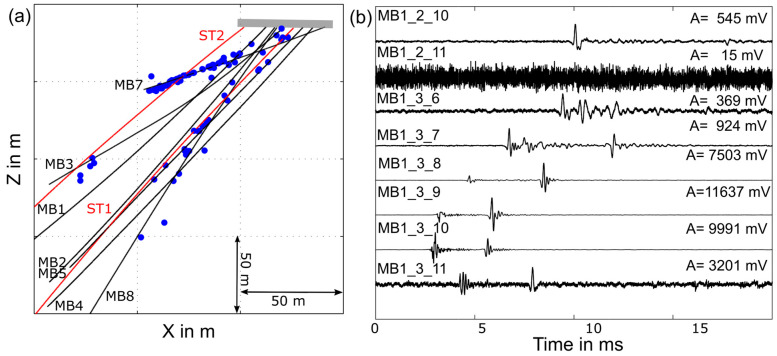
Data of example seismic events. Shown in (**a**) is the seismic activity from 10 to 28 September 2021. The activity is correlated to the newly cemented boreholes MB5, MB7, and MB8, i.e., to picoseismicity triggered by the hydration and temperature input from cementation. Monitoring boreholes are shown as black lines; production boreholes are shown as red lines; the tunnel is shown as a grey line. In (**b**) we show the waveforms of a typical seismic event of this time period and recorded on AE sensors of two boreholes. The waveforms are normalized; the maximum amplitude of each trace is given on the right. The network is capable of recording very small seismic events with good signal-to-noise ratios. As discussed in the text, AE sensors are not-calibrated; therefore, the absolute energy (magnitude) of the event is uncertain at this time and subject to further analysis.

All seismic sensors were installed at their designated position in the monitoring boreholes. Seismic waves covering the full frequency range from 0.08 Hz to above 100 kHz are successfully recorded in the ongoing stimulation experiments. Nonetheless, a significant number of sensors installed in the monitoring boreholes failed. One geophone in MB3 is non-functional, presumably because of damage during pre-cementing the guide shoe. The first three guide shoes were pre-cemented with slightly expanding cement slurry. A total of 10 single components of triaxial accelerometers and 21 AE sensors do not record data. Furthermore, 14 out of these 21 sensors are installed in the deep borehole sections of borehole MB1 and MB4. We speculate that the preamplifiers of these sensors were damaged due to an unforeseen delay in the cementation. Whereas all other boreholes were cemented within 20 days after instrumentation, the sensor chains in MB 1 and MB3 stayed 5 months and 3 months, respectively, in the formation water. The delay was caused by logistical problems. We speculate that these sensors failed over time due to water leakage into the preamplifier. Due to the redundant design (we expected some sensor failure due to difficulties and challenges described in the first paragraph), with more sensors installed than theoretically necessary, we find for the high-resolution zone that despite seismic sensor failing, the sensor density is sufficient to record high-quality data (Figure 10). Small picoseismic events speculated to be in the magnitude range M < −5 (the magnitude calculation is still ongoing) were recorded on more than 5 sensors from several tens of meters’ distance from the injection borehole. The resolution for small seismic events in the deeper parts of the Bedretto reservoir is diminished due to the sensors failing in the deep parts of MB1 and MB4.

### 3.7. Active Seismics

#### 3.7.1. Active Seismics: Objectives and Requirements

We aim at sampling the rock volume with active sources in order to monitor changes in the rock volume’s elastic moduli or in the coda wave in time and space. Active seismic methods are based on the artificial emission of seismic waves from a source that is recorded by receivers at a certain distance. Temporal–spatial changes and variance in scattering are used to constrain, e.g., fracture density over time or to locate newly generated fractures. The dominant wavelengths of the signals emitted by the source define which structures can be imaged. In BRP, we are mostly interested in monitoring the development of fractures from 0.1 m to 10 m, and accordingly need a source in the frequency range of 500 Hz to 60 kHz. Furthermore, the source must be suitable for cemented boreholes. In order to fulfill the criteria, we chose ultrasonic transmitters. Sparkers, the most common source in borehole seismics and suitable to generate signals with frequencies up to approximately 5 kHz, are for use in open boreholes only and, therefore, not applicable in the BRP monitoring boreholes. Furthermore, the emitted signal of ultrasonic transmitters is more repeatable than sparker signals.

#### 3.7.2. Active Seismics: Implementation

To actively probe the experimental volume, we installed twelve ultrasonic transmitters manufactured by GMuG. The transmitters were positioned around the high-resolution zone of the experimental volume at borehole depths between 40 m and 167 m, ensuring that the central volume has a dense ray coverage.

The disc-shaped devices have a height of 26 mm and can be placed next to the central tube. We installed five transmitters of type GMuG-Tr70 with a dominant frequency of 29 kHz and seven transmitters of type GMuG-TR50 with a dominant frequency of 40 kHz. GMuG-Tr70 comes with a disc diameter of 70 mm, which is the largest diameter we can fit next to the central tube. The large diameter ensures stronger signals. GMuG-Tr50 comes with a disc diameter of 50 mm and accordingly a slightly weaker signal but emits signals with higher-frequency content. The brass housing comes with two arms for clamping to allow a firm connection to the rod system even if friction due to flowing water or cement is present. Transmitters are clamped to the central pipe using 3D-printed holders and brackets.

#### 3.7.3. Active Seismics: Results

**Figure 11:**
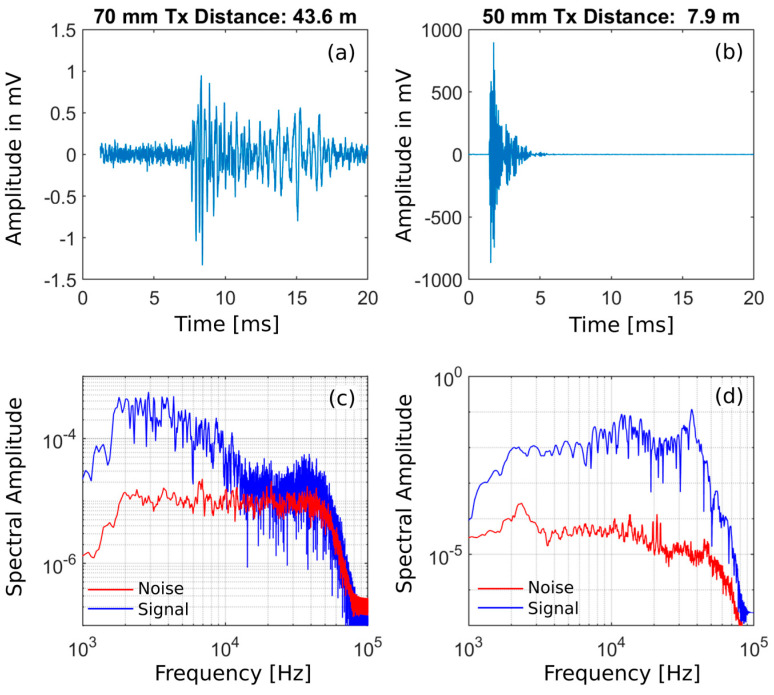
Data of example ultrasonic transmitters in BRP. In (**a**) the raw waveform recording of transmitter signal GMuG-Tr70 over 43.6 m distance is shown (1738 stacks). The corresponding spectrum is shown in (**c**). In (**b**), the waveform of transmitter GMuG-Tr50 over 7.9 m distance is shown (1524 stacks). The corresponding spectrum is shown in (**d**). Due to intrinsic damping, higher frequencies are transmitted for short distances only. In (**d**), frequencies above 70 kHz are visible, whereas in (**c**) only frequencies up to 15 kHz are recorded above the noise floor.

The ultrasonic transmitters are detected in the Bedretto granite for up to 81 m so the design allows crossing ray paths of different source–receiver pairs in the central volume. For the longest distances, only frequencies f < 10 kHz are transmitted due to intrinsic damping along the ray path. Over short source–receiver distances, active signals with energy content f > 70 kHz are recorded (Figure 11). 

### 3.8. Installation

#### 3.8.1. Installation: Objectives and Requirements

A detailed installation concept was developed to ensure smooth installation and to address the following risks: (1) fiber-optic cables may not be bent or squeezed hard; (2) installation and guidance systems must be fixed securely at all times to prevent uncontrolled sliding into the borehole; and (3) always keep track of the exact rod length and realize complete documentation in order to stay informed of which installation step is next to ensure correct sensor positioning. The instrumentation was executed following a detailed instrumentation plan containing the exact position of different sensors, clamps, and centralizers defined by borehole depth relative to the central installation tubing (distance from the socket).

#### 3.8.2. Installation: Implementation

The monitoring boreholes were instrumented in February 2020 (MB2), June and July 2020 (MB1 and MB4), September 2020 (MB3), and July 2021 (MB5, MB7, and MB8). Installation was spread over several time intervals for (a) logistical reasons, and (b) to allow for careful re-assessment of the installation procedure in between. For example, as discussed above, experiences from the second installation allowed changes in top plug design and fiber-optic cable design. Before installation could start, all logging and characterization campaigns within the boreholes had to be finalized. 

Installation was conducted in a two-shift system with four persons per shift. The installation took approximately three to five days per borehole. The available space at the borehole mouth allowed one rod to be installed at a time. Each rod holds one centralizer with cable holders and sensors, where applicable. Two persons insured at all times that the system was secured and clamped at all times in order to prevent uncontrolled sliding into the boreholes (four-eye control). After the installation of instruments on one rod was finished, the instrumentation and guidance system was lowered into the borehole until the next rod could be placed. The whole system was secured during the installation by an electrical winch (HIT-TRAC 16E manufactured by Habegger, Trubschachen, Switzerland). Fixation was realized by four rock anchors above the borehole and in the tunnel roof. For redundant security, a steel block was clamped to the rod, which ensured that the system could not slide into the borehole. The installation comes with long cables wound on cable drums. In order to ensure smooth and untangled cable installation, we installed all cable drums on rods fixed inside a framework. In this way, the cable drums were secured and could be unrolled easily. Cables were kept straight and separated until they reached the installation point (centralizer with cable clamp). During unwinding, each drum was under supervision of a person to spot irregularities in unwinding early. In regular intervals, cables were tested for damage using red light for fiber-optic cables and a multimeter for electrical cables. We find that the controlled unwinding of cables as well as regular testing of cables during the installation is crucial, especially for long boreholes and fiber-optic cables.

### 3.9. Cementation

#### 3.9.1. Cementation: Objectives and Requirements

In the cementation we aim at maintaining—as much as possible—rock integrity, protecting sensors and limiting cross-hole flow and communication between fracture zones along the wellbore. The slurry design for sealing the monitoring boreholes has to fulfill several requirements to achieve these goals and ensure optimal installation of the monitoring equipment. The number of sensors installed and cables routed through the well head pose a particular challenge for the cementing operations. We defined the following functional requirements for sealing: (a) Inflow of water from over-pressurized fracture zones has to be counteracted and stopped by the cement column weight to minimize cement dilution and enable good consistent cement quality; (b) loss of cement to intersecting fracture sets has to be eliminated or minimized to avoid cementation of pre-existing shear zones in the reservoir and to avoid the flow of cement into neighboring wells. Cement loss must be prevented, especially in the upper fracture zones because here cement loss would result in a cement head several tens of meters below the wellhead and leave the borehole partially uncemented. (c) Flow of fluids along the borehole, i.e., channels in the cement, e.g., due to imperfect sealing along cables or along (micro-)annuli must be prevented. Artificial pathways extending over a significant length of the borehole would impact the experimental stimulation and circulation campaigns planned in the reservoir volume; (d) equipment such as acoustic emission and strain sensors as well as geophones must be well coupled to the rock volume to provide good data quality; (e) air bubbles (>5 mm in diameter) have to be avoided to reduce local attenuation of seismic energy by dispersion; (f) the reflection coefficient between rock and cement should be kept as small as realistically possible to avoid additional dispersion; (g) the pH value of the cement should be as low as possible to control decomposition of rubber seals and cable material; (h) temperatures must remain below 60 °C at all times, including during curing; (i) cement reaction should be completed within a reasonable time (months) to avoid any transient behavior influencing the experiment; and finally (j) there must be no expansion of cement during hardening to avoid unintentional fracturing of the surrounding rock mass. We achieve the functional requirements by designing the following slurry properties: viscosity, hydration heat, density, setting time. Please note that controlling the hydration heat is not only important to control temperature development but also the formation of annuli by avoiding thermal shrinkage. As a minimum target, all sensors should be embedded in good cement and, therefore, at a minimum, the top of the cement should be situated above the highest sensor.

#### 3.9.2. Cementation: Implementation

We developed the cement composition specifically for the BRP monitoring boreholes with support of SIKA Schweiz AG, Zurich, Switzerland, a company with expertise and a product portfolio of cement additives for construction purposes. In the development, various CEM III furnace slag cement (Holcim Modero 3B) slurries were pre-tested on-site (Table 5) to identify a cement system that features a stable rheology and can be pumped through the central tubing with acceptable pressure losses at sufficiently high flow rates. A minimum flow rate of 30 L/min was identified as optimal during testing. This allows for cementation of the deepest wellbore (MB1) within 6 h, which is the maximum available pumping time for the selected cement system.

CEM III furnace slag cement features a low-hydration heat while developing a high strength. As a low shrinkage cement, leaking along the micro annuli can be mitigated and good sensor coupling is realized. We decided against adding expanding agents such as MgO or CaO, which are used to overcome cement shrinkage and the forming of micro-annuli. While micro-annuli can be avoided very effectively with these additives, these metal oxides have the tendency to react over very long time periods; hence, they can impose stress–strain changes to the solidified cement for a longer period (months to years). The CEM III cement system features a lower pH than standard CEM I cements. The pH drops quickly to pH 12 subsequent to the initial hydration phase. We discarded other pH-reducing additives such as carbonates due to their impact on the final cement compressive strength. We added bentonite clay and SIKA underwater (UW) compound as a stabilizer to eliminate forming of any free water during the setting of the cement slurry. Free water can lead to channels within the cement, in particular at the high side of deviated or horizontal wells.

The density of the cement slurry was chosen after in-field testing based on the following considerations: The density of the slurry has to be high enough to control inflow from the reservoir, while to the same extent pumping should be continuous and as fast as possible to limit the mixing zone. Mixing takes place as long as the cement column pressure is lower than the formation pressure during cementing across a specific inflow zone. Furthermore, a cement with high density was preferable to minimize the impedance contrast between solid cement and granitic rock, which reduces the energy scattering of the seismic wave.

We deployed a high-viscosity cement system, in order to force good displacement of the water column when pumping the cement slurry. The high viscosity minimizes the mixing with the displaced water column above the cement and reduces the likelihood of residual water inclusions in the cement. In order to keep the high viscosity cement slurry pumpable, a liquid fluidizer (Sikament S12) was added to the slurry. The fluidizer enhances the shear thinning behavior of the cement slurry and thereby reduces the flowing pressures at higher shear rate, i.e., the flow pressure while pumping through the tubing or annulus. Likewise, at low shear rates, i.e., in fractures, the viscosity remains high and limits the leak-off into the fractures. The pumpability of the slurry was tested upfront by simulating the wellbore tubing with 48 m of pipes and hoses with an inner diameter similar to the installed tubing.

In total, five cement slurries were tested for the BRP project. On-site testing is important to test the cement composition under real conditions, e.g., with on-site water used in the final sealing operation and in the on-site temperature regime. From each test slurry, a test sample of about 20 L was taken and stored in a barrel. Periodically, the Marsh funnel time was measured to identify the onset of slurry thickening and thereby define the maximum pumping time available. The pressure losses caused by flowing the slurry through the 1″ test tube were measured in situ. Test slurry 0 served during testing as a baseline slurry without additives. In test slurry 1 and test slurry 2, we tested small water–cement ratios (*w*/*c*). In test slurry 2, Sikament S12 was added to maintain good pumpability and bentonite was used as a rheology stabilizer. About 1.5 vol% of free water was observed after 6 h of rest period when investigating test slurry 2, which is too much for the BRP application, where we aim ideally for no settling at all. In test slurry 3, we used UW compound additives as an additional stabilizer which was very effective and limited foaming of the slurry, i.e., it reduced the amount of bubbles significantly and increased the slurry viscosity considerably. In addition, the concentration of Sikament S12 was increased in slurry 3, which worked out well as a fluidizer. The pressure losses in the 1″ test tube could be limited to 5.2 kPa/m. The resulting frictional loss is 1.5 MPa for 300 m tubing length, which is acceptable with respect to the utilized pumping equipment. Finally, in test slurry 4 we investigated the option to work with a very viscous cement system that can be utilized to control larger fracture apertures. The very high viscosity resulted in 200 mbar/m pressure losses at 30 L/min flow rate. For a 300 m long tubing, this would result in 6 MPa frictional losses, which might exceed the available pumping capacity. Consequently, test slurry 4 can only be pumped at lower flow rates and is of interest for specific sections of the well with high leak potential. In order to limit the leak-off to open fractures, we added, were appropriate, lost circulation material (LCM) to the slurry. LCMs are granular materials with a defined grain size distribution. We chose carbonate sand from the sand blasting industry with a grain size distribution of 90 µm to 500 µm. The grain size distribution was selected in accordance to the estimated fracture aperture. Due to the slightly higher solid content, the viscosity of a slurry is slightly increasing with LCM. It was discussed and agreed with the cement manufacturer (Holcim) that 5% carbonate additive will not affect the chemical reactions/setting behavior of the cement system. Carbonate sand is soft enough to minimize wear/damage to the plunger of the cement pump; hence, the carbonate sand can be mixed into the cement slurry directly.

For the sealing operation we composed a sequence (lead and tail slurry, excess volume) of slurry 3 and slurry 4 individually for each borehole (Table 6) taking into account potential loss zones or other well-specific requirements. MB7 was cemented with slurry 3 solely because this borehole had no signs of open fracture sets and did not cross the main shear zones. For all other boreholes, we selected the high-viscosity test slurry 4 as the lead slurry with LCM. For MB3, a very viscous cement pill of 200 l (corresponding to 10 m borehole length) was pumped before the lead slurry in order to fill the sandy debris in the bottom part of this well. (Before, we demonstrated with an on-site pre-test that a cement system with higher UW compound concentration (2 wt%) can be poured through a water column and forms solid cement below the water level). The amount of LCM was 5 vol% in the early cementation operations and was increased in the lead slurry of MB1 due to the high risk of leakage into production borehole ST2. The amount of LCM was also increased in the lead slurry of MB5 and MB8, the later cementations, based on our good experience of the LCM cement with respect to the fracture sealing capacity as well as with respect to the pumpability when sealing the other boreholes.

We controlled the inflow of mountain water into the boreholes during cementation by reducing the acting pore pressure levels. For this, we allowed the reservoir to drain for about 48 h. The drainage reduced the maximum overpressures observed in the deepest fracture interval at 289 m along hole depth in MB1 from 4 MPa to about 0.65 MPa. As tested beforehand, the pressure decrease had a sufficiently long recovery time to allow for cement curing. In order to stop the water inflow by the cement slurry overpressure, a sufficient cement column height above the specific fracture zone pressure level has to be reached during pumping. A density of 1.8 g/cm^3^ assures that even with the top of cement at the minimum level, the water inflow of the uppermost fracture zone can be controlled. At the same time, the overpressure at the lowest fracture zone for all wells is maintained sufficiently high. Still, the cement slurry should accept a certain amount of dilution by water inflow from the specific fracture zone without compromising any of the required functionalities. A larger mixing zone, expected due to the described dilution effect, was circulated out. We prepared accordingly adequate excess volumes of cement for each borehole. The cement quality of the returns was monitored by measuring the density and when the minimum density of 1.6 g/cm^3^ was reached, the cementing operation was stopped and the well was shut in.

A three-fold strategy was deployed to minimize or stop leakage of cement in the existing fracture sets and control the short-cut between ST2 and MB1. First, the high viscosity of the cement system (130 cP) limits the leak off into small fractures because higher differential pressure is required to flow considerable volumes of cement in fractures with small apertures. Once the slurry comes at rest, the fast gelation and forming of a particular gel strength supports this process. A pressure differential above the flowing pressure is required to break the gel and restore the flow of cement slurry into the fracture. This measure alone can control small fracture apertures (ten to one hundred micrometers). Larger apertures were controlled with LCM: the grain size distribution is adapted to the expected fracture aperture to allow some of the grains in the slurry to flow into the fracture and then block the fracture off by clogging in conjunction with the higher viscosity fluid.

The largest probability for cement loss was estimated for monitoring borehole MB1, where a pre-existing fracture creates a short-cut to borehole ST2. The aperture of the shortcut between ST2 and MB1 was estimated based on a transmissivity value derived from hydraulic cross hole testing. Utilizing a simple parallel plate model, the reported transmissivity of the short cut (10 − 4 m^2^/s) equals a fracture aperture of 0.5 mm. A high viscosity cement slurry alone can result in considerable cement loss (up to 1500 L) in such a fracture considering the long setting time of the cement (min. 4 h) and the maximum differential pressure at the shortcut of 1.5 MPa; therefore, LCM is needed. We added LCM with a grain size distribution between 90 and 500 µm, which is sufficient to treat fractures with apertures up to 1.5 mm, i.e., we designed the slurry as a precaution for a fracture apparatus larger than estimated. Note, LCM systems are effective for slightly larger apertures than the grain size due to the conglomeration of particles when flowing into small, irregular openings.

#### 3.9.3. Cementation: Results

Sealing the boreholes to restore the integrity of the rock volume as much as possible was successful, but an individual design for some boreholes was needed. Through borehole-specific adjustments, the cementation of MB1 (cross-cut to ST2) and MB3 (significant sand deposition at borehole foot) was performed without further problems.

In MB4, cement losses were observed; i.e., no cement returned to the surface although the full access volume has been pumped during the operations. The top of the cement in MB4 was found at 30 m AHD (along hole depth). This means that about 1.5 m^3^ of cement slurry leaked off during the operation. The losses indicated that the initial concept of a lead cement slurry with LCM (slurry 4) followed by less viscous cement without LCM (slurry 3) cannot fully control cement leak-off into the fracture sets. A likely reason for the underperformance of the LCM cement in MB4 could be the limited height of the initial LCM cement column within the wellbore. A total of 1000 1 were pumped initially, corresponding to 50 m of wellbore length (along the hole). If the cement column does not exceed the pore pressure at depth sufficiently, the leak-off into the fracture set is limited; hence, the specific fracture set does not clog due to the placement of the LCM material.

Based on the experiences with MB4, we postponed the cementation of MB1 in order to rethink and modify the LCM cementation concept. The new concept introduced, on top of the LCM lead cement slurry, a larger LCM cement excess volume pumped at the end. The volume of the tail slurry was relatively small (Table 6). MB1 was cemented without any obvious cement losses, despite an identified, highly conductive shortcut from MB1 to ST2. Although we cannot independently assess the effect of the tail slurry, we speculate that in the case of insufficient sealing of deeper fracture sets by the lead slurry with LCM, the late LCM cement would stop cement losses. No traces of cement were identified in the outflow of ST2 during the cementing operations. In order to pump larger volumes of the LCM tail slurry, the flow rate had to be reduced to adapt to the larger frictional pressure losses when pumping. Data from the geomechanical sensors recorded during different stages of the cementation of MB1 were used to (a) monitor the cementation progress and (b) develop a retrospective and integrated approach to analyze borehole integrity [35]. Based on the good results in cementing MB1, we continued using the LCM tail slurry for the cementation of MB5 and MB8.

## 4. Discussion and Conclusions

We evaluated the challenges and risks associated with the realization of multi-disciplinary, high-resolution monitoring networks in hectometer-scale underground experiments where long boreholes (100 m to 300 m in length) need to be instrumented with a variety of different sensors. The overall complexity of the installation in hectometer-scale experiments is significantly higher than for decameter-scale experiments due to longer boreholes. The most dominant technical challenges are:

- Higher uncertainties in borehole geometry and borehole quality due to the higher stresses;
- Difficulties due to borehole roughness to guide instrumentation safely to the anticipated depth without blockage or sensor/cable damage;
- Damage to sensors due to water intrusion and high pressures in long boreholes;
- Sealing the boreholes without unintended channeling, fracturing owing to expanding cement, cement loss, or uncontrolled inflow of mountain water diminishing the cement quality.

In the framework of the Bedretto Reservoir Project, an experimental volume of approximately 100 m × 300 m × 100 m was instrumented in boreholes of 101 m to 304 m in length, but extensive technical development was necessary. The following goals were successfully achieved:

- The novel instrumentation setup allowed the installation of different sensors in the same borehole and the combined monitoring of seismo-hydromechanical processes. The monitoring network has been in use since autumn 2021 for a series of stimulation experiments.
- All instruments were successfully brought into the boreholes and to their final position despite substantial breakout zones. Blockage of the system was successfully prevented.
- Boreholes were successfully sealed using a purpose-made slurry. Challenges (10 m sand column in MB3; conductive short-cut between MB1 and ST2) were controlled with LCM cement slurry and sealed off.
- High-quality data were successfully recorded from each sensor type. The recording goals were reached inside the high-resolution monitoring volume.

Difficulties encountered and failures confirm, on the other hand, the severe challenges in realizing high-resolution monitoring in hectometer-scale experiments:

- Curvature in borehole trajectories and clogging of boreholes (MB3 and MB4) in later time periods required altering the sensor geometry several times, which was logistically a problem and caused delays.
- While sealing successfully prevented drainage through the boreholes, we observed that some cables caused drainage. Most importantly, the multi-fiber cables used for installing FBGs caused a small amount of water drainage through the interior of the cable. In addition, three coaxial cables of one accelerometer leaked water, while the specially designed multi-coaxial cable remained impermeable. Whether this low-level drainage is influencing the experiments is subject of further investigations.
- A significant number of sensors are not recording data (Table 1). FBG sensors were lost due to damage in cables and seismic sensors failed due to electronics damaged by pressure and humidity. Redundancy in the network design allowed to compensate for broken sensors to some extent and the monitoring goals were achieved within the high-resolution monitoring volume. The monitoring is diminished in the deep parts of the experiment volume owing to broken sensors.
- The overall logistics were challenging. Moreover, individual pieces were handy, the total sum of material added up to several tons, which had to be transported into the tunnel and installed into the borehole by hand.

We conclude that the following key elements were especially important for the realization:

- Break up of drilling and instrumentation into several campaigns. This allowed us to adjust the borehole geometry and the sensor positions based on logging results and gave us time to react to problems encountered and adjust the plans.
- A centralizer with integrated cable clamp for cable protection and guidance.
- Individual design of the cementing operations for each borehole to address borehole-specific difficulties and the sealing of loss zones.
- Cementable tube pore pressure sensor, for true pore pressure measurements in cemented boreholes and simultaneous installation with FBG sensors.

Overall, instrumenting hectometer-scale experiments with diverse, scientific monitoring systems is expensive, both in an organizational and financial manner. The BRP monitoring network demonstrates that complex, high-resolution monitoring is possible. Nonetheless, decameter-scale experiments are more controllable; therefore, the benefits of stepping-up to hectometer scale should be carefully evaluated before such an experiment. We encourage groups interested in decameter- or hectometer-scale underground experiments to implement monitoring systems capable of conducting imaging small-scale processes.

The instrumentation procedure developed for BRP will be implemented in future ETH projects, e.g., the upcoming Bedretto Earthquake Nucleation Project. To which extent the approaches developed in the framework of BRP are suitable to be used in full-scale surface boreholes will be investigated. We speculate that some components such as the centralizer with integrated cable holder or the tube pore pressure sensor are in theory suitable for much deeper borehole depths.

## Figures and Tables

**Table 1:** Overview of equipment installed in long monitoring boreholes for the BRP experiment. The monitoring network is accompanied by additional sensors installed in short boreholes or platforms in the main tunnel. Both the number of installed sensors is given as well as the number of working sensors in early 2022 (in brackets). Details on the sensors including the custom-made sensor adaptions and reasons for sensor failure are discussed in Section 3.4, Section 3.5, Section 3.6 and Section 3.7.

Sensors	No.	Monitoring Borehole	Sensor Length in mm	Sensor Diameter in mm	Custom Made
Rod System					
Central pipe	763	1, 3, 4, 5, 7, 8	3000	33.7	Yes
Centralizer	>800	1, 3, 4, 5, 7, 8	526	157.1	Yes
Frontshoe	6	1, 3, 4, 5, 7, 8	955	141.3	Yes
Endpacker	3	1, 3, 4	1490.5	168	Yes
Multipacker	7 (6)	2	1000	88	Yes
Seismology					
Geophone	6 (5)	1, 3, 4, 5, 7, 8	350	56	Yes
Wilcoxon acc.	4 (2)	1, 4, 5, 7	110	49.9	Yes
Endevco acc.	3 (3)	1, 3, 4	110	49.9	Yes
AE sensor	60 (38)	1, 3, 4, 5, 7, 8	117	30	Yes
Geomechanics					
Pressure sensor	7 (6)	2	110	30	No
CTPP	4 (4)	5, 8	540	145 (123)	Yes
FBG	70 (58)	1, 5, 7, 8	1070	17.5	No
Fiber-optic cable	full length	1, 3, 4, 5, 7, 8	-	-	No
Ap. Geophysics					
Ult. transmitter	12 (12)	1, 3, 4, 5, 7, 8	100	50/70	Yes

**Table 3:** Overview about boreholes drilled in the Bedretto Reservoir Project. Maximum deviation describes the deviation from straight trajectories. Borehole bending occurred as shown in Figure 1.

Borehole	Length in m	Mean Azimuth	Mean Dip	Max. Deviation in m	Distance to STI11 in m	Borehole Diam. in mm
ST1	404.8	227.10	49.15	20.9	-	216
ST2	350.9	224.68	41.79	25.1	32.9–47.0	216
MB1	304.2	227.56°	40.3	24.4	19.9–45.4	165
MB2	221.7	227.66	48.0	7.7	13.4–16.5	101
MB3	189.6	225.84	32.56	24.8	6.7–45.3	165
MB4	253.3	226.46	45.43	15.5	12.8–16.3	165
MB5	221.8	225.63	44.42	9.4	6.3–13.4	165
MB7	101.1	252.11	23.62	11.2	13.2–35.7	165
MB8	252.0	219.36	52.41	12.9	11.1–25.6	165

**Table 4:** Overview of seismic sensors.

	Sensor	Sensor Type	Manu-Facturer	Frequency Range in Hz	Digitizer	Location
Regional Seismology	STS2	Broadband Seismometer	Quanterra	0.08–50	Nanometri. Centaur	Tunnel
Strong Motion Seism.	Episensor	Accelero-meter	Kinematrics	DC-200	Nanometri. Centaur	Tunnel
Microseismicity	MBAS Single	227.56°	Geotomog.	100–1000	Nanometri.	5 m boreh.
	GS-100 Hz	227.66	IMS	100–1000	Centaur	MB
	PSS-56	225.84	Avalon	15–1600		MB
Nano-seismicity	ACC-3A25	Piezoelec. Accelerom.	IMS	50–25,000	GMuG	MB
	ACC-45A19			50–6000	AEsystem	MB
Pico-seismicity	GMuG-Ma-Blc-30–35	AE Sensor	GMuG	1000–50,000	GMuG AEsystem	MB

**Table 5:** Cement properties of slurries tested by the BRP for borehole sealing.

	*w*/*c*	Bentonite	UW Compound	Sikament S12	Density in g/cm^3^	Thicken. Time	Marsh Funnel t	Viscosity in cP *	Free Water in vol%	P Loss ** in kPa/m
Slurry 0 (base line)	0.6	-	-	-	1.57	>6 h	45 s	33	Not measured	2.8
Slurry 1	0.5	-	-	0.8 wt%	1.63	>6 h	44 s	32	10	-
Slurry 2	0.5	0.5 wt%	-	1.0 wt%	1.52	6 h	47 s	36	1.5	3.4
Slurry 3	0.5	0.4 wt%	0.4 wt%	1.7 wt%	1.72	>4 h	105 s	130	0.0	5.3
Slurry 4	0.5	0.6 wt%	0.7 wt%	1.2 wt%	1.8	>4 h	140 s	200	0.0	20.3

* Viscosity estimated from Marsh funnel time; ** 1″ tubing, 30 L/min.

**Table 6:** Cement volumes and pumping sequences in chronological order.

Borehole	Pre-Lead Slurry	Lead Slurry	Tail Slurry	Excess Volume
MB3	200 l UW cement *	1000 l Slurry 4 + LCM	3000 l Slurry 3	800 l Slurry 3
MB4	-	1000 l Slurry 4 + LCM	4500 l Slurry 3	1000 l Slurry 3
MB1 **	-	2600 l Slurry 4 + LCM	2000 l Slurry 3	2400 l Slurry 4 + LCM
MB7	-	-	2000 l Slurry 3	700 l Slurry 3
MB5	-	2000 l Slurry 4 + LCM	2100 l Slurry 3	2700 l Slurry 4 + LCM
MB8	-	2700 l Slurry 4 + LCM	2000 l Slurry 3 + LCM	2800 l Slurry 4 + LCM

* UW cement is based on slurry 4 with 2 wt% of UW compound. ** Risk of leakage to neighboring well required a high volume of LCM cement.

## Data Availability

Not applicable.
